# CD300LF^+^ microglia impede the neuroinflammation following traumatic brain injury by inhibiting STING pathway

**DOI:** 10.1111/cns.14824

**Published:** 2024-07-04

**Authors:** Zhichao Lu, Zongheng Liu, Chenxing Wang, Rui Jiang, Ziheng Wang, Weiquan Liao, Wei Wang, Jianfeng Chen, Xingjia Zhu, Jingwei Zhao, Qianqian Liu, Yang Yang, Peipei Gong

**Affiliations:** ^1^ Department of Neurosurgery Affiliated Hospital of Nantong University, Medical School of Nantong University Nantong Jiangsu China; ^2^ Neuro‐Microscopy and Minimally Invasive Translational Medicine Innovation Center Affiliated Hospital of Nantong University Nantong Jiangsu China; ^3^ Jiangsu Medical Innovation Centre, Neurological Disease Diagnosis and Treatment Center Affiliated Hospital of Nantong University Nantong Jiangsu China; ^4^ Research Center of Clinical Medicine Affiliated Hospital of Nantong University Nantong Jiangsu China; ^5^ Department of Neurosurgery, Zhejiang Provincial Hospital of Chinese Medicine The First Affiliated Hospital of Zhejiang Chinese Medical University Hangzhou China; ^6^ Department of Biobank Affiliated Hospital of Nantong University Nantong Jiangsu China; ^7^ Department of Pathology Affiliated Hospital of Nantong University Nantong Jiangsu China; ^8^ Department of Orthopedics and Traumatology Wuxi TCM Hospital Affiliated to Nanjing University of Chinese Medicine Wuxi Jiangsu China; ^9^ Department of General Surgery, Shanghai Key Laboratory of Biliary Tract Disease Research, Research Institute of Biliary Tract Disease Xinhua Hospital Affiliated to Shanghai Jiao Tong University School of Medicine Shanghai China; ^10^ Department of Neurosurgery Wuxi Taihu Hosptial Wuxi China

**Keywords:** CD300LF, microglia, neuroinflammation, single‐cell RNA sequencing, STING, traumatic brain injury

## Abstract

**Introduction:**

The diversity in microglial phenotypes and functions following traumatic brain injury (TBI) is poorly characterized. The aim of this study was to explore precise targets for improving the prognosis of TBI patients from a microglial perspective.

**Objectives:**

To assess whether the prognosis of TBI can be improved by modulating microglia function.

**Results:**

In CD300LF‐deficient mice, we observed an increase in glial cell proliferation, more extensive neuronal loss, and worsened neurological function post‐TBI. Transcriptomic comparisons between CD300LF‐positive and CD300LF‐negative microglia illuminated that the neuroprotective role of CD300LF is principally mediated by the inhibition of the STING signaling pathway. In addition, this protective effect can be augmented using the STING pathway inhibitor C‐176.

**Conclusions:**

Our research indicates that CD300LF reduces neuroinflammation and promotes neurological recovery after TBI, and that microglia are integral to the protective effects of CD300LF in this context. In summary, our findings highlight CD300LF as a critical molecular regulator modulating the adverse actions of microglia following acute brain injury and propose a novel therapeutic approach to enhance outcomes for patients with TBI.

## Graphical abstract

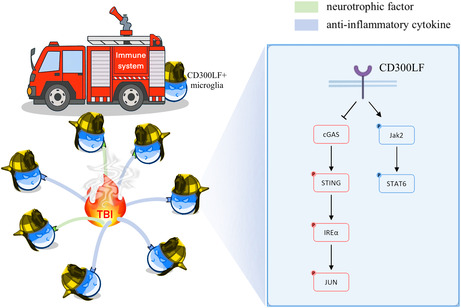

## 1 INTRODUCTION

Traumatic brain injury (TBI) contributes to lifelong motor, cognitive, and behavioral abnormalities, and is a high‐risk factor for the progression of other neurodegenerative disorders such as Alzheimer's and Parkinson's diseases.^1^ Neuroinflammation, as an important secondary injury process following TBI, plays an important role in both the acute and chronic phases, leading to progressive brain edema, synaptic disruption, neuronal apoptosis, and deterioration of neurological function.^2^ The activated microglia are considered to be the primary initiators of and participants in the inflammatory response in the central nervous system (CNS).^3^ Microglia can be categorized into different cell subtypes based on their morphology, density, electrophysiological properties, and immune‐molecular markers on the cell surface. Advances in single‐cell transcriptome sequencing have facilitated the study of microglial heterogeneity in various diseases.^4^, ^5^, ^6^ It has been discovered that microglia exhibit distinct genetic profiles responsive to changes in the CNS microenvironment post‐injury.^7^ For example, microglia may express CD16/32, CD86, and nod‐like receptor (NLR) family member NLRP3, producing inflammatory cytokines like iNOS and IL‐1β that aggravate injury. Conversely, microglia can also express CD206 Arg‐1, and Ym1, releasing anti‐inflammatory cytokines like IL‐10 and TGF‐β1, which help clear hematomas and cellular debris and mitigate neuroinflammation.^8^, ^9^ Therefore, understanding microglia heterogeneity offers valuable insight into the pathogenesis of secondary CNS injuries. Furthermore, targeting the activation of microglia towards neuroprotective subtypes presents a promising therapeutic strategy for TBI treatment.

CD300LF is a type I integral membrane protein prevalently expressed on the surface of myeloid cells, including macrophages, dendritic cells, neutrophils, and B cells. It functions as a receptor molecule, orchestrating immune responses through its binding to phospholipids on dead and dying cells and subsequently triggering pro‐inflammatory or anti‐inflammatory signals, depending on the specific milieu.^10^ CD300LF was recognized as the main physiological receptor for murine norovirus and mediated the inflammatory response and cytotoxicity.^11^ Recent studies have shown that microglia expressing CD300LF counteract malaria‐induced blood–brain barrier disruption, cerebral edema, and cerebral hemorrhage by inhibiting neuroinflammatory responses.^12^ Nevertheless, the specific location and functions of the CD300LF‐positive microglia subpopulation following brain injury remain to be elucidated.^13^

In this study, we integrated tissue samples from TBI patients with single‐cell sequencing data from mice to pinpoint and delineate a distinct subset of microglia, identified as CD300LF‐positive, in the post‐TBI brain. By using flow cytometry targeting different brain regions, we characterized the distribution of CD300LF^+^ microglia in the post‐TBI brain. Data from CD300LF knockout mice suggest that CD300LF plays a pivotal role in alleviating neuroinflammation and preserving neurological function post‐injury, underlining that microglial involvement is indispensable for CD300LF's protective impact on TBI. By comparing the transcriptomic profiles of CD300LF^+^ microglia and CD300LF^−^ microglia, we have revealed that CD300LF exerts neuroprotective functions by inhibiting the activity of the STING signaling pathway. Correspondingly, the STING signaling pathway's selective inhibitor, C‐176, has demonstrated promising neuroprotective properties in the context of TBI. In summary, our research presents evidence for the beneficial role of the CD300LF‐positive microglia subset in attenuating neuroinflammation and enhancing neuroprotection after TBI. Augmenting the population of CD300LF‐positive microglia post‐TBI could contribute to decreasing the mortality and morbidity associated with TBI and offer novel insights for clinical application.

## 2 MATERIALS AND METHODS

### 2.1 Single‐cell analysis

The expression matrices of GSE160763 were acquired from the GEO database.^14^ Quality control and dimensionality reduction were carried out using the “Seurat” package. In the initial quality control step, Seurat objects were created, and cells expressing fewer than 200 genes were filtered out. Genes expressed in fewer than 3 cells were also excluded. The gene expression profiles of the remaining cells were then normalized, and the top 2000 highly variable genes were identified using the vst method for each sample. All genes were scaled, and principal component analysis was conducted. Unsupervised clustering was performed on the cells, and the top 20 principal components were visualized using UMAP. Cell‐type annotation was performed using the “singleR” package. Differentially expressed genes (DEGs) between Cd300lf+ and Cd300lf− microglia were extracted (|log2 fold‐change (FC)| > 1 and *p* < 0.05). Subsequently, Kyoto Encyclopedia of Genes and Genomes (KEGG) and Gene Ontology (GO) analyses were conducted to investigate the enriched pathways and biological processes of the DEGs using the “clusterProfiler” package in R. In addition, gene set enrichment analysis (GSEA) was performed using the R software packages “org.Hs.eg.db,” “clusterProfiler,” and “enrichplot” to identify enriched pathways between the Cd300lf+ and Cd300lf− microglia groups.

### 2.2 Sample collection and evaluation of patients with TBI

The study protocol was approved by the Ethics Committee of Affiliated Hospital of Nantong University (Approval No. 2023‐K187‐01). Brain tissue samples with contusions were obtained from patients who were admitted to the neurosurgery department of the Affiliated Hospital of Nantong University for traumatic brain injury (TBI) surgery (Table S1). These samples were then screened based on the specified criteria, which included the inclusion of TBI patients with Glasgow Coma Scale (GCS) scores equal to or less than 12. The GCS is utilized to assign scores ranging from 3 to 15, with lower scores indicating more severe brain injury. The exclusion criteria employed in this study encompassed individuals under the age of 18, those with penetrating head injuries, individuals with brain tumors, patients who succumbed within 24 h, individuals who did not undergo surgical removal of hematoma within 24 h of injury, patients with multiple injuries, those with a simplified injury rating exceeding 2 in extracranial body regions, individuals with infectious diseases upon admission, patients who received blood transfusions within 24 h of injury, individuals currently taking immunosuppressive medications, patients undergoing treatment with steroids, individuals with a history of severe organ system insufficiency, those with an immunocompromised status, or pregnant individuals. Patients who declined to participate in the study or their legally authorized representatives were likewise excluded. Prior to the surgical procedure, these individuals furnished written informed consent in the presence of a witness, adhering to established protocols for neurosurgical practitioners. The consent form employed for this particular study was acquired prior to the surgery. Brain tissue from the cleared contusion area was procured during the surgical intervention. Human samples were obtained in strict adherence to the protocol sanctioned by the Institutional Review Board of Nantong University Hospital. Fresh brain tissue samples were procured from the vicinity surrounding the hematoma, which is commonly referred to as the injury zone. The corresponding tissue homogenates were obtained and subsequently subjected to analysis utilizing flow cytometry, immunofluorescence, and polymerase chain reaction (PCR) techniques.

### 2.3 Experimental animals

Cd300lf‐KO mice (Cat. NO. NM‐KO‐200096) were purchased from Shanghai Model Organisms Center, Inc. Four‐week‐old C57BL/6 mice were obtained from the Nantong University Animal Experiment Center in Jiangsu, China. The mice were housed in a specific‐pathogen‐free facility, with no more than five animals per cage, and subjected to standardized light–dark cycle conditions. They had ad libitum access to food and water. The vivarium was maintained at a constant temperature of 21°C and a humidity level of 50%–60%. All mice were used in experiments when they were approximately 6 months old. All experimental procedures were in accordance with the Guidelines on the Care and Use of Laboratory Animals. The animal research conducted in this study was approved by the Nantong University Laboratory Animal Ethics Committee (Approval No. IACUC20231125‐1001).

### 2.4 Establishment of TBI models

As we previously described,^15^, ^16^ the TBI model was induced in mice. The mice were anesthetized with isoflurane gas and placed on a stereotaxic apparatus. Anesthesia was administered via 1%–3% isoflurane inhalation, and ventilation was provided using oxygen‐enriched air (20% O_2_:80% air) through a nasal cone. A midline incision was made on the scalp, and a bone window of 4.0 mm in length and 2.0 mm in width was created 1.0 mm posterior to the herringbone suture and 2.0 mm lateral to the midsagittal line. Flush the area with saline to remove bone fragments and ensure the integrity of the dura mater. The brain is tilted at an angle of 15° and perpendicular to the impactor (3 mm diameter tip; RWD LifeScience, China). The impactor parameters for CCI were impact speed, 3.5 m/s; deformation depth, 1.30 mm; duration, 400 ms. After the impact, rinse the area with saline again and stop the bleeding by using a sterile cotton swab. Seal the bone window with bone wax, suture the scalp, and use erythromycin ointment to prevent infection. Place the mice on a heating pad until they recover from anesthesia. For the sham operation group, craniotomy was performed to expose the dura mater, but no impact was performed.

### 2.5 Evaluation of lesion volume

First, the length and width of the surface of the lesion area were measured, and then the lesion volume was briefly estimated (*V*, mm^3^). The calculation formula is *V* = (length × width)^2^ × 1/2.

### 2.6 Measurement of brain edema

Brain edema was detected by the wet‐dry weight method. After euthanasia, the mice were quickly removed from their brains and placed in a precooled PBS solution to remove excess blood and pollutants. Drain the surface of the brain and weigh the weight of the injured and contralateral cerebral cortex. The cortex was then dehydrated in an oven at 100–110°C for more than 24 h and re‐weighed. The percentage of water content in brain tissue is calculated as follows: (wet weight‐dry weight)/wet weight × 100.

### 2.7 Quantitative real‐time polymerase chain reaction (qRT‐PCR)

Human samples were taken from the contusion brain tissue of patients who underwent hematoma clearance on the first day after TBI, and animal samples were taken from the injured side of the cerebral hemisphere. As we described before,^16^ following tissue disruption and homogenization in 1 mL of QIAzol, along with 200 μL of chloroform and vortexing for 15 s, RNA extraction from the brain was conducted using QIAzol (Qiagen, Germany). To achieve phase separation, the samples were allowed to sit at room temperature for 10 min before being centrifuged for 10 min at 12,000 *g* at 4°C. The upper layer, which contained the RNA, was meticulously transferred to a separate tube and precipitated with an equal volume of isopropanol. After centrifugation for 10 min at 8000 *g* at 4°C, ethanol was eliminated from the samples, and they were subsequently air‐dried. The RNA pellet was reconstituted in 20 μL of RNase‐free dH_2_O. The concentration of RNA was determined using NanoDrop. For reverse transcription, 0.75 mg of RNA (diluted in a total volume of 40 μL of dH_2_O) was incubated at 70°C for 10 min with 5 μL of random hexamers (Biomers, Germany). The samples were then cooled on ice, and a master mixture containing 0.5 μL of reverse transcriptase (Promega, Germany), 0.5 μL of RNase Inhibitor (RiboLock, Thermo Fisher Scientific, Germany), 2 μL of dNTPs (Genaxxon, Germany), and 12 μL of reverse transcriptase buffer (Promega, Germany) was added. The samples were initially incubated at ambient temperature for a duration of 10 min and subsequently transferred to a water bath set at 42°C for a period of 45 min. After this, the samples underwent an additional incubation of 3 min, followed by placement on ice for preservation and future utilization.

The primers utilized in this study were developed through the utilization of the NCBI primer blast program, which is affiliated with the National Center for Biotechnology Information in the United States. The corresponding sequences were acquired by employing the NCBI nucleotide search tool, GenBank. The primer blast tool was employed to fine‐tune parameters in order to optimize the output of the polymerase chain reaction (PCR) for the in‐house light cycler. To summarize, the range of the PCR product size was established as 70 to 140, the primer melting temperature (Tm) was determined as 60°C ± 3°C, no specific preference was given to the exon junction span, and the appropriate organism was chosen. The primer pair exhibiting the highest Tm, self‐complementarity, and self‐3′ complementarity was selected, and the primer pairs were subsequently verified for specificity towards the target gene using the primer blast tool. Prior to their utilization in experiments, the primers were validated by conducting a test sample alongside the appropriate controls (samples lacking RNA and samples lacking reverse transcriptase) to confirm the Ct value, thereby ensuring primer selectivity and functionality. Detailed primer sequences for each gene examined can be found in Table S2.

In each sample, RNA (2 μg) was subjected to reverse transcription using the TMRT kit (Takara Bio Inc., Japan). Subsequently, a quantitative reverse transcription polymerase chain reaction was conducted on an ABI QuantStudio5 Q5 real‐time PCR System (Thermo Fisher Scientific, USA) employing SYBR Green (Roche, Germany). The 2^−ΔΔCt^ method was employed to determine and standardize relative mRNA expression with respect to GAPDH expression.

### 2.8 Flow cytometry

Human samples were taken from the contusion brain tissue of patients who underwent hematoma clearance on the first day after TBI, and animal samples were taken from the injured side of the cerebral hemisphere. The tissue was chopped up and treated with collagenase IV and deoxyribonuclease I at 37°C for 30 min. After being rinsed with phosphate‐buffered saline (PBS), the removal of myelin debris was achieved through centrifugation in a solution of 30% percoll (Sigma Aldrich), followed by another rinse with PBS and resuspension in a solution containing 1% bovine serum albumin. Subsequently, fluorescent‐conjugated antibodies were introduced to the cell suspension and allowed to undergo staining while being kept on ice for a period of 30 min. Following the application of surface marker labeling, the cells were subsequently fixed in a fixing buffer for a duration of 20 min and subsequently underwent two washes with permeabilization buffer. Intracellular molecule‐specific antibodies were subsequently introduced and allowed to stain the cells for a period of 45 min. Following the staining process, the cells were washed and suspended in a flow cytometry buffer.

The data collected using a FACS Aria III flow cytometer was analyzed using FlowJo software. Flow cytometry gating strategy of human and mice brain cellular lineages, including microglia (CD45^low^CD11b^+^), astrocyte (CD45^−^CD11b^−^GFAP^+^), neuron (CD45^−^CD11b^−^NeuN^+^). The table of essential resources includes all antibodies utilized in the flow cytometry staining procedure can be found in Table S3.

### 2.9 Western blot

Human samples were taken from contusion brain tissue of patients who underwent hematoma clearance on the first day after TBI, and animal samples were taken from the injured side of the cerebral hemisphere. Brain homogenates were extracted as previously described.^15^ After centrifugation, the brain tissue pellet was thoroughly mixed with cell lysis buffer containing a mixture of 1% protease inhibitor and 1% phosphatase inhibitor, and then lysis was promoted by ultrasound. After standing for 30 min, centrifuge and collect the supernatant. All the above operations are performed on ice. Total protein concentration was assessed using a bicinchoninic acid protein assay kit. The proteins are boiled after thorough mixing with the loading buffer. Subsequently, sodium dodecyl sulfate‐polyacrylamide gel electrophoresis was performed on a 10% gel to effectively separate proteins of different molecular weights. The proteins were then transferred to a 0.45 μm polyvinylidene difluoride membrane. After a 2‐h blocking procedure using 5% bovine serum albumin solution, the membrane was incubated with primary antibodies overnight. Subsequently, the membrane was incubated with secondary antibodies for 2 h at room temperature, and protein bands were observed using ECL (Billerica Millipore, USA). Protein bands were then characterized and quantified using a ChemiDoc detection device (Bio‐Rad, USA) and analyzed using ImageJ software (National Institutes of Health, USA). Specific details of the antibodies used can be found in Table S3.

### 2.10 Immunostaining

After anesthetizing the mice as described above, pericardial perfusion was performed using 0.9% saline solution and 4% paraformaldehyde. Brains were then extracted and fixed in 4% paraformaldehyde overnight at room temperature. The brain tissue was then dehydrated using 30% sucrose solution. Prepare 12 μm sections using a cryotome (Thermo Fisher Scientific USA) and store at −20°C until needed. Preincubate with 5% blocking serum in 0.3% TritonX‐100 for 1 h, then incubate with primary antibody overnight at 4°C. TUNEL kits (C1089, Beyotime, China) were used to detect the level of apoptosis in the injured area, which we performed according to the manufacturer's protocol. After rinsing three times in PBS, sections were exposed to a mixture of secondary antibodies for 2 h at room temperature. Sections were then examined using a fluorescence microscope (DM5000B; Leica). Each mouse chose five slices near the center of the impact site (between Bregma‐1.5 mm and ‐2.5 mm). The areas photographed by immunofluorescence were the perifocal cortex and the dentate gyrus of the hippocampus. Six images were taken in each area. The cell coverage area, number and average intensity per mm^2^ were calculated by Image J analysis software. All the statistical calculations were carried out by researchers who did not know about the experimental design. Antibodies used for immunofluorescence assays are detailed in Table S3.

### 2.11 Sholl analysis

Z‐stack images were acquired on a Leica Thunder 3D Assay inverted fluorescence microscope with 40× magnification. Microglia were selected based on IBA1 staining and astrocytes were selected based on GFAP staining. The ImageJ plugin “Sholl Analysis” was used to draw concentric circles centered on the nucleus and spaced 5 μm apart and to calculate measurements including soma volume, total process length and the number of intersections per radius.

### 2.12 Primary microglia cell culture

Briefly, after we removed the brains of neonatal mice, we carefully removed the meninges and collected the cortex. The tissue was cut into small pieces and digested with 0.25% trypsin at 37°C for 20 min, and then the cells were cultured in 25 cm^2^ culture flasks covered with 0.01% PLL. Cells were grown in F‐12 medium containing 10% fetal bovine serum for 2 weeks, with medium changes every 3 days. Once cells reach confluence, place the flask on a rotary shaker at 37°C and 180 rpm for 30 min to detach microglia. The isolated microglia were then transferred to culture dishes for further study. The purity of isolated primary microglia was confirmed to be >97% using ionized calcium‐binding adapter molecule 1 (IBA1) labeling. Regarding cell transfection, Cd300lf mouse overexpression plasmid (CAT#: MR204865, ORIGENE) and Cd300lf mouse shRNA plasmid (CAT#: TR514771, ORIGENE) was transfected into primary microglia according to the manufacturer's guidelines.

### 2.13 Phagocytosis assay

Immunoglobin G antibodies labeled with fluorescein isothiocyanate‐coated beads (Cayman, USA) were added to microglia‐containing dishes at a concentration of 1:1000. After 8 h of co‐culture, cells were washed 3 times with PBS solution and examined under a fluorescence microscope (DM5000B; Leica, Germany). Three visual fields were randomly selected and the average intensity of IgG positive cells in each visual field was calculated to detect the phagocytic ability of microglia. The average intensity was calculated by researchers who did not know about the experimental design.

### 2.14 Microglia depletion in vivo

For pharmacological ablation, C57BL/6J mice were fed with chow containing the CSF1R inhibitor PLX5622. The PLX5622 compound was formulated in AIN‐76A chow by the company (SYSE Bio‐tech Co., Ltd) at a dose of 1200 p.p.m (1200 mg PLX5622 per kilogram chow). Mice were fed PLX5622 or control AIN‐76A (without PLX5622) for 3 weeks before undergoing TBI surgery. Mice were maintained on the same diet until sacrifice.

### 2.15 Drug administration

The STING antagonist C‐176 (MedChemExpress, cat # HY‐112906) was dissolved in a stock solution containing 5% DMSO and 95% corn oil (Sigma) and then diluted in PBS (5% v/v). Mice were injected intraperitoneally every day with C‐176 or vehicle (PBS containing 5% stock solution) at a dose of 10 mg/kg after TBI until they were killed.

### 2.16 Modified neurological severity score (mNSS)

As we used before, to assess the extent of neurological deficits in mice, mNSS was assessed on days 1, 7, and 14 after TBI.^16^ mNSS includes some behavioral indicators related to the nervous system, such as limb movement, sensation, coordination and reflexes. Each reflex loss or abnormal response is assigned a score. Thus, on a scale of 0–18, a score of 0 represents normal neurological function with no deficits, while a score of 18 represents severe neurological deficits.

### 2.17 Open field test (ORF)

The open‐field test is a method of evaluating autonomous motor behavior, exploratory behavior, and stress in mice. The device consists of an open rectangular box with detectors on all sides (40 × 40 × 30 cm, Accuscan Instruments, USA). Mice were first acclimated to the experimental environment for 1 h. During the experiment, mice were placed in the center of the box and allowed to explore freely for 30 min. Total distance traveled was calculated using Versmax analysis software. Open field testing was performed 14 days after TBI surgery. All experiments and data analysis were performed in a blinded manner.

### 2.18 Rotarod test

Rotarod test was performed as previously described.^15^ Mice were trained on an accelerated (5–30 rpm) rotarod for 3 days. During the test phase, mice were placed on an accelerating rotary rod with the speed increasing from 5 rpm to 30 rpm over 5 min. The latency of mice to fall from the rod was recorded, and each mouse was trained for 5 trials (each trial run for 5 min, rest for 5 min). The final score is the average latency of the 5 falls of the experimental mice. Rotarod testing was performed 1 day before TBI and 1 and 14 days after TBI. All experiments and data analyses were performed in a blinded manner.

### 2.19 Y‐maze test

Y‐maze is a test of discriminative learning, working memory and reference memory applied to mice. It consists of three identical long arms (30 × 10 × 20 cm) at 120° intervals. Mice were acclimated to the experimental environment for 1 h prior to the experiment. Mice were then placed in the central area and allowed to explore the maze freely for 5 min while their movements were recorded by a camera placed above the maze. When mice were fully in the long arm, they were considered to have made a choice. Three different consecutive choices were considered alternating. The percentage of alternation was calculated using the following formula: Total alternates/(Total choices − 2) × 100. Experiments were performed 14 days after TBI. All experiments and data analyses were performed in a blinded manner.

### 2.20 Statistical analysis

The data were analyzed utilizing GraphPad Prism 9.14 and subsequently expressed as the mean ± standard error of the mean. We used the Shapiro–Wilk test to assess the normality of the distribution of continuous variables. All data are tested for normality, and data that does not show a normal/Gaussian distribution is analyzed by nonparametric equivalents. Comparisons were made using the unpaired Student's *t*‐test, one‐way analysis of variance (ANOVA), and two‐way ANOVA. A *p*‐value of 0.05 was deemed to indicate statistical significance. Each experiment was replicated a minimum of three times.

## 3 RESULTS

### 3.1 Expression of CD300LF is upregulated in microglia after TBI

**FIGURE 1:**
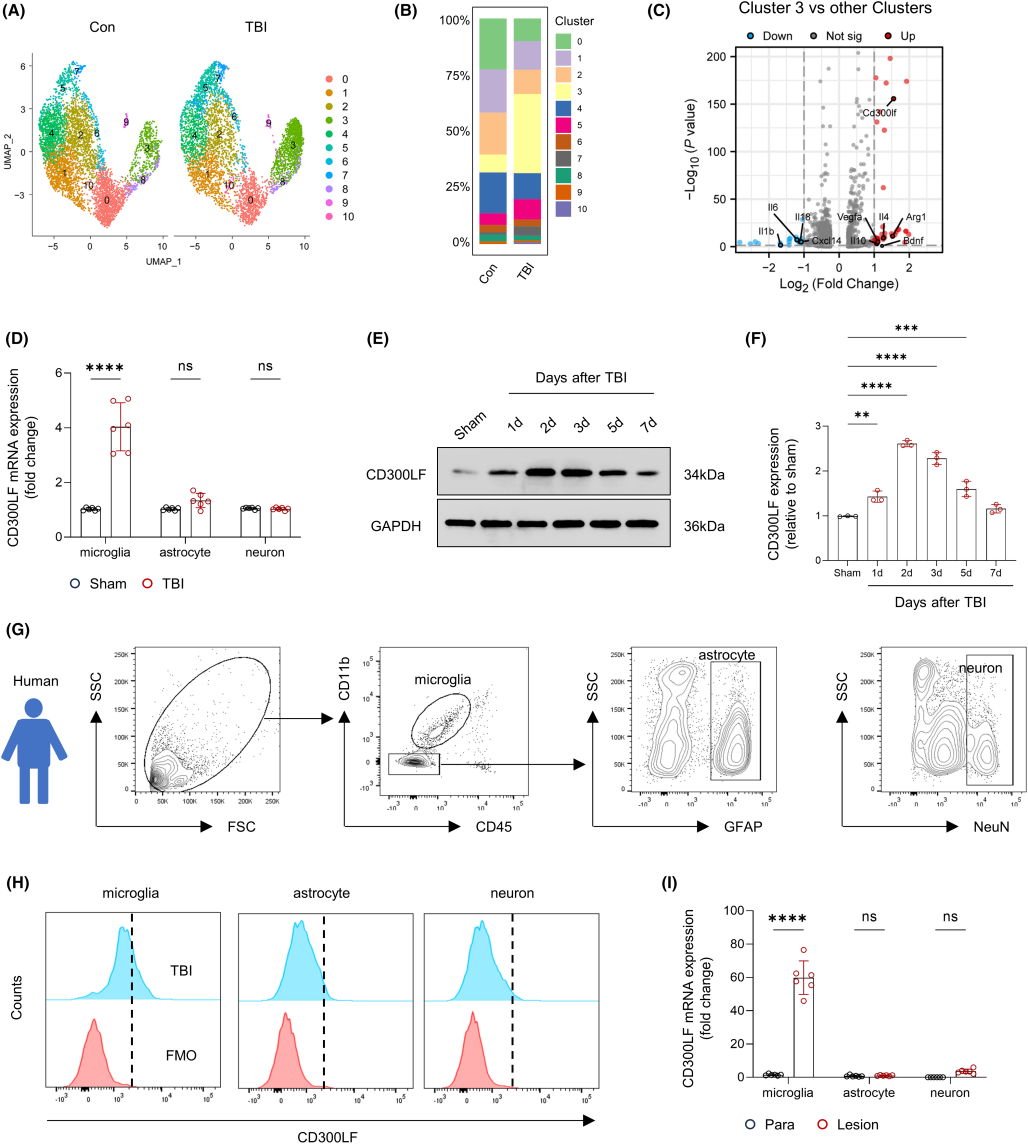
CD300LF expression is upregulated in microglia after TBI. (A) Representative UMAP results of single‐cell sequencing data of microglia before and after TBI. (B) Quantitative analysis was performed for the percentage of microglia subpopulations before and after TBI. (C) Volcano plot shows DEGs in Cluster3 microglia versus other Cluster microglia. (D) PCR assays were performed to detect the expression of Cd300lf in microglia, astrocytes and neurons before and after TBI in mice. (E, F) The proteins of the injured lateral cerebral hemisphere at different time points in the sham group as well as in the TBI group were extracted and subjected to protein detection by western blots and analyzed for relative CD300LF quantification. (G) Flow cytometry strategies for detecting immune cells in brain tissue obtained surgically after human TBI. (H, I) Detection of CD300LF expression in microglia, astrocytes, and neurons in brain tissue from human injury area. H: Flow cytometry assay and I: PCR assay. *n* = 6/group (D, I) and *n* = 3/group (F). Data are presented as mean ± SD. **p < 0.01, ***p < 0.001, ****p < 0.0001. Statistical analyses were performed using two‐tailed unpaired Student's *t* test (D, I) and one‐way ANOVA followed by Tukey post hoc test (F).

We first investigated changes in the proportion of cell types in brain tissue before and after TBI using single‐cell sequencing data published by Witcher et al.^14^ The results of unbiased clustering of the sequencing results revealed that cells in brain tissue can be classified into eight cell types. After TBI, we mostly observed an expansion in the population numbers of microglia, monocytes, oligodendrocytes and astrocytes as well as a decrease in the population numbers of other cell types including neurons and endothelial cells (Figure S1A,B). Further analysis showed that CD300LF was upregulated in microglia (Figure 1C, Figure S1C).

To further characterize this population of CD300LF^+^ microglia, we visualized single‐cell sequencing data of microglia before and after TBI in isolation. The results of UMAP showed a significant population expansion of Cluster3 microglia after TBI (Figure 1A,B). Considering that CD300LF is predominantly expressed in microglia after TBI, we compared the transcriptome of Cluster3 microglia with that of the remaining Clusters microglia. The results of the volcano plot prompt us that the transcriptome of Cluster3 microglia exhibits up‐regulation of inflammation‐regulating cytokines genes such as *Cd300lf*, *Il4*, *Arg1* together with the neurotrophins *Bdnf*, whereas the expression of pro‐inflammatory cytokines such as *Il1b*, *Il6* as well as the neutrophil chemokine *Cxcl14* are significantly down‐regulated in Cluster3 (Figure 1C, Figure S2A, Table S4).

To determine the expression profile of CD300LF in TBI, we sorted different cell types and measured its RNA expression in microglia, astrocytes, and neurons before and after TBI. The results of qRT‐PCR suggested that the expression of *Cd300lf* was significantly elevated in microglia but not astrocytes or neurons after TBI (Figure 1D). Consistently, the western‐blot analysis revealed a rapid elevation of CD300LF after TBI: At 24 h after TBI, CD300LF levels were dramatically elevated, which peaked at 48–72 h after TBI and then declined to control levels (Figure 1E,F).

We next quantified the preferred infiltration locations of CD300LF^+^ microglia after TBI. In the brains of sham group mice, CD300LF microglia were evenly distributed in all regions (Figure S3A,B). After TBI, CD300LF microglia were inclined to infiltrate around the injured area of the cerebral cortex, with a moderate increase seen in the hippocampus, but not in other brain regions (Figure S3C,D).

We went further to perform similar analysis in injured area and para‐injured area of brain tissues from 6 patients with TBI who received surgery. The results of flow cytometry showed that microglia expressed more CD300LF compared to other cell types after TBI (Figure 1G,H). In light of murine TBI model, we observed significant up‐regulation of CD300LF transcription in microglia of brain tissue in the injured area (Figure 1D,I). Thus, up‐regulation of CD300LF in microglia cells is trans‐species.

### 3.2 CD300LF inhibits excessive glial cell responses in the injured area after TBI

**FIGURE 2:**
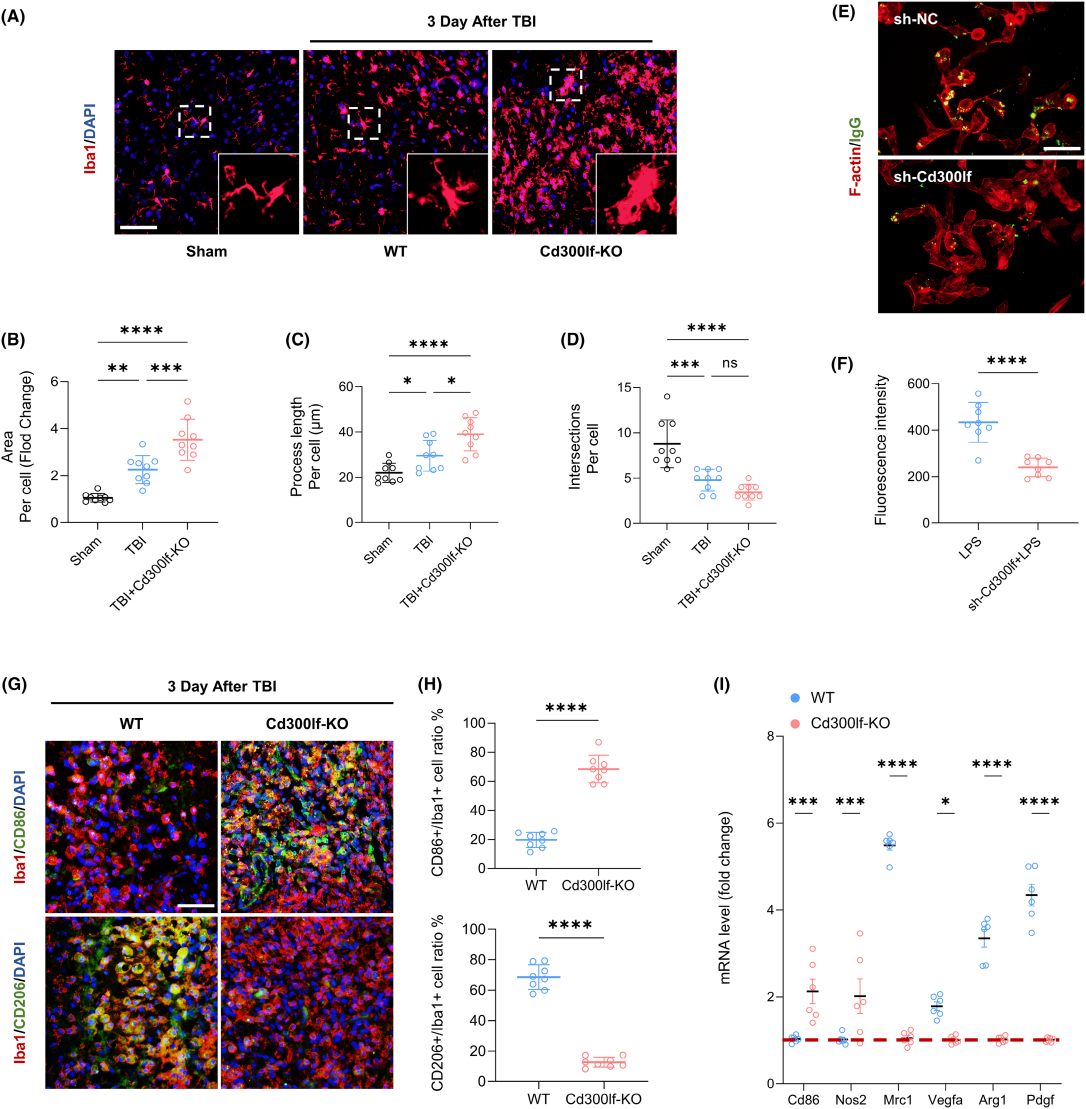
CD300LF inhibits excessive microglia responses after TBI. (A) Representative microglia immunofluorescence images after TBI in Sham group, WT group and Cd300lf‐KO group. (B–D) Sholl analysis for microglia. B: mean microglia area, C: mean microglia process length and D: mean microglia interactions. (E, F) The cellular phagocytosis capacity of primary microglia before and after knockdown of Cd300lf was detected, and the fluorescence intensity of IgG fluorescent particles phagocytosed by microglia was quantitatively analyzed. (G, H) Representative immunofluorescence images of CD86 and CD206 in the WT and Cd300lf‐KO groups after TBI and quantitative statistics of cell proportions. (I) PCR assays were performed to detect the expression of microglia‐associated activation markers in the WT and KO groups after TBI, and relative quantitative statistics were performed. *n* = 8/group (A–H); *n* = 6/group (I). Data are presented as mean ± SD. *p < 0.05, **p < 0.01, ***p < 0.001, ****p < 0.0001. Statistical analyses were performed using two‐tailed unpaired Student's *t* test (F, H, I) and one‐way ANOVA followed by Tukey post hoc test (B–D).

To determine the potential role of CD300LF in TBI, we investigated the effects of CD300LF blockade on microglia function in TBI mice. We used wild‐type (WT) mice as well as CD300LF knockout (KO) mice for TBI modeling. Inflammatory microglia have been reported to exhibit enlarged cytosol and synaptic gyrus, indicative of a pathological state.^17^ We therefore quantitatively analyzed the morphology of Iba‐1^+^ microglia around the injured area at day 3 after TBI. The sholl analysis showed that WT mice had less area and process length of microglia infiltrated around the injured area and more intersections with concentric circles than those infiltrated in the injured area of KO mice (Figure 2A–D). Interestingly, after interfering with CD300LF expression in primary microglia in vitro, microglia showed a significant decrease in phagocytosis, indicating decreased expression of CD300LF induced a compromise ability of microglia in clearing debris and dead cells after TBI (Figure 2E,F). We then examined the expression of functional markers in the microglia infiltrated in the injured area. Interestingly, the microglia infiltrated in the injured area of WT mice were predominantly expressing CD206 as compared to TBI mice in KO, whereas CD86 positive microglia were mainly clustered around the injured area of KO mice (Figure 2G,H). We next examined the expression of common markers associated with inflammatory regulation. The results of qRT‐PCR revealed that the transcript levels of inflammation‐suppressing and tissue‐repair‐associated markers such as *Mrc1*, *Vegfa*, *Arg1*, and *Pdgf* were higher in WT TBI mice, whereas in the KO mice, we found a significant enhancement in the transcription of pro‐inflammatory markers *Cd86* and *Nos2* (Figure 2I).

**FIGURE 3:**
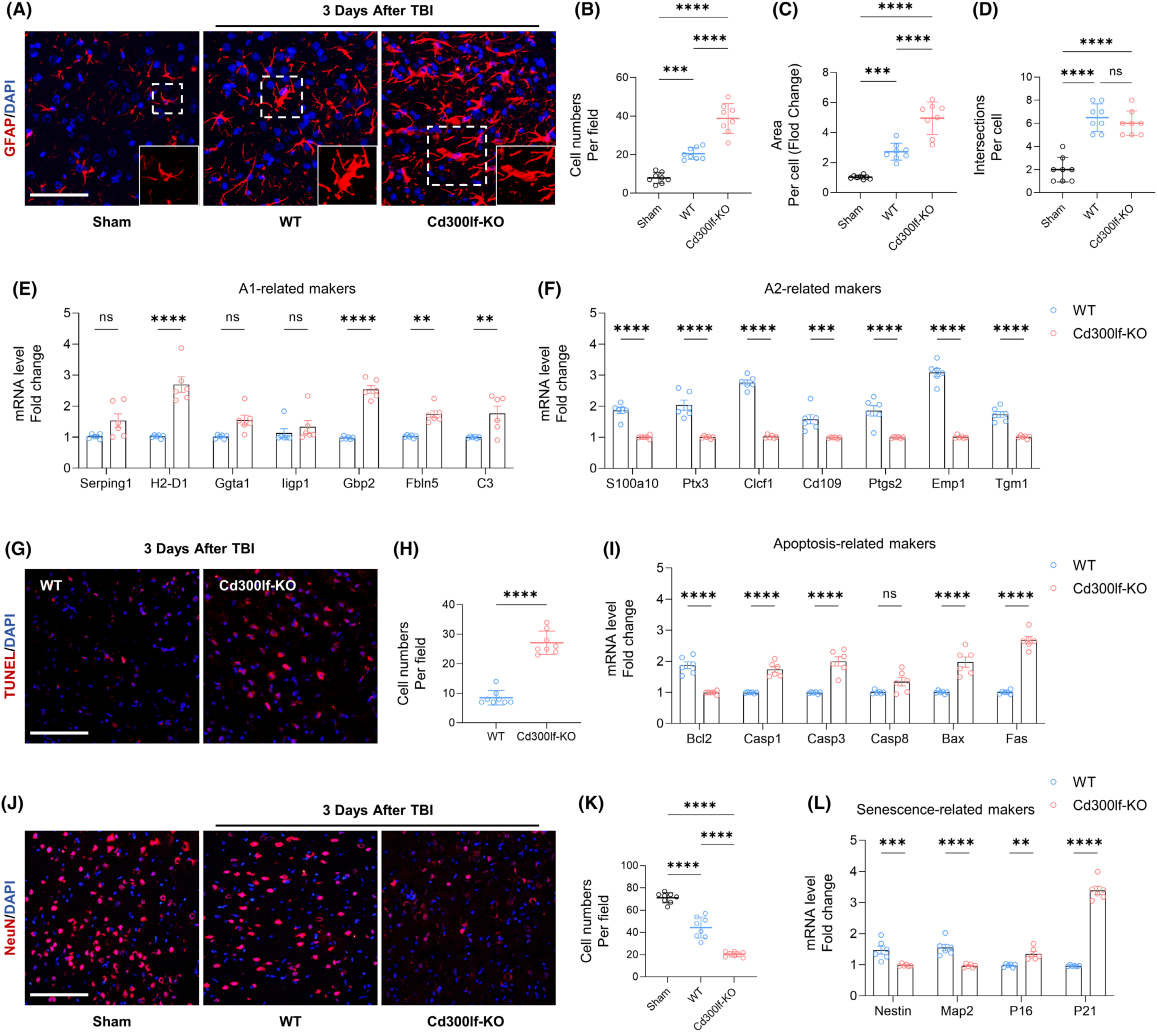
CD300LF inhibits astrocyte overreaction after TBI and protects neurons. (A) Representative astrocytes immunofluorescence images after TBI in Sham group, WT group and Cd300lf‐KO group. (B–D) Sholl analysis for astrocytes. B: cell numbers/per field, C: mean astrocytes area and D: mean astrocyte interactions. (E, F) PCR assay to detect astrocyte activation‐related markers after TBI. E: A1‐related markers and F: A2‐related markers. (G, H) Representative TUNEL fluorescence images in the WT and KO groups after TBI and quantitative analysis. (I) PCR assay to detect apoptosis‐related markers after TBI. (J, K) Representative immunofluorescence images of neurons in the WT and KO groups after TBI and quantitative analysis. (L) PCR assay to detect senescence‐related markers after TBI. *n* = 6/8 per group. Data are presented as mean ± SD. **p < 0.01, ***p < 0.001, ****p < 0.0001. Statistical analyses were performed using two‐tailed unpaired Student's *t* test (E, F, H, I, L) and one‐way ANOVA followed by Tukey post hoc test (B–D, K).

The function of astrocytes, as an important component of the neuroimmune microenvironment after TBI, is critical to the prognosis of TBI patients. The results of Sholl analysis similarly showed that the number and area of astrocytes around the injured area in WT mice were smaller than those around the injured area in KO mice whereas there were more intersections of infiltrating astrocytes and concentric circles in WT mice (Figure 3A–D).

### 3.3 CD300LF protects damaged neurons after TBI

The purpose of modulating the excessive inflammatory response around the injured area is to reduce neuronal death.^18^ For this purpose, we examined the level of neuronal apoptosis in the injured area after TBI in WT mice and KO mice by using TUNEL assay kit. Without the protection of CD300LF, the number of TUNEL‐positive cells around the injured area of KO mice was significantly increased, along with significant elevation of five apoptosis‐related genes, demonstrating the inhibitory effect of CD300LF on apoptosis (Figure 3G–I). We also examined neuron numbers around the injured area. Consistent with the apoptotic state, KO mice exhibited a significant decrease in neuron numbers around the injured area compared to WT mice, as well as a significant decrease in the levels of the markers of newborn neurons, *Nestin* and *Map2*, whereas transcripts of markers indicative of cellular senescence, such as *P16* and *P21*, were significantly up regulated. Taken together, we hypothesized that CD300LF protects damaged neurons by modulating the inflammatory state and inhibiting apoptosis in the CNS after TBI (Figure 3J–L).

### 3.4 CD300LF attenuates leukocyte infiltration after TBI and promotes the production of immunomodulatory cytokines thereby protecting neurological function in mice

**FIGURE 4:**
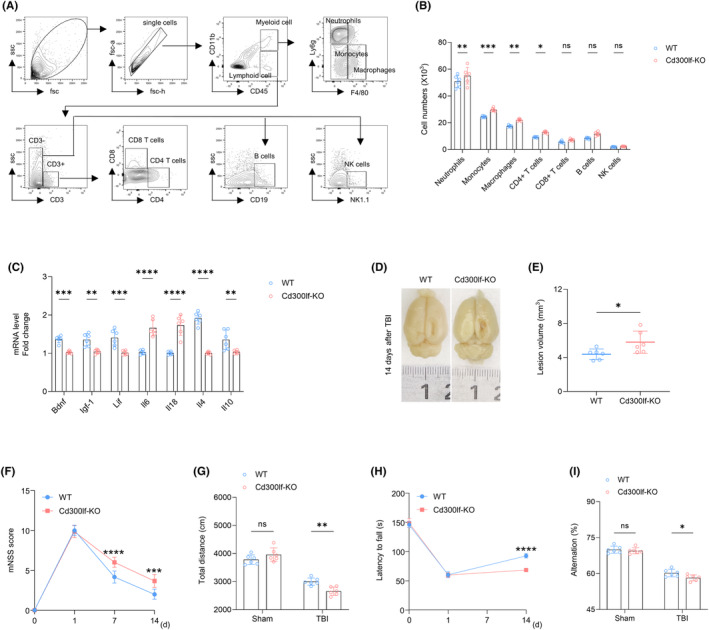
CD300LF inhibits the inflammatory response and promotes neurological recovery after TBI. (A, B) Flow cytometry strategies for detecting immune cells infiltrating the CNS after TBI and quantitative counts of various types of immune cells. (C) PCR assay to detect inflammation‐related markers after TBI. (D, E) Images of representative injury areas in the WT and KO groups after TBI. The lesion volume was quantitatively counted. (F–I) Results of behavioral testing in the WT and KO groups after TBI. F: mNSS score, G: ORF, H: Rotarod test and I: Y‐maze. *n* = 6 per group. Data are presented as mean ± SD. *p < 0.05, **p < 0.01, ***p < 0.001, ****p < 0.0001. Statistical analyses were performed using two‐tailed unpaired Student's *t* test (B, C, E) and two‐way ANOVA followed by Tukey post hoc test (F–I).

We measured brain infiltrating leukocytes in the brains of TBI mice using flow cytometry. At day 3 after TBI, we found that CD300LF decreased the number of brain‐infiltrating neutrophils (CD45^hi^CD11b^+^Ly6G^+^), monocytes (CD45^hi^CD11b^+^Ly6G^−^F4/80^−^), macrophages (CD45^hi^CD11b^+^Ly6G^−^F4/80^+^), and CD4+ T cells (CD45^hi^CD3^+^CD4^+^). However, there was no reduction in the number of CD8+ T cells (CD45^hi^CD3^+^CD8^+^), B cells (CD45^hi^CD3^−^CD19^+^), and NK cells (CD45^hi^CD3^−^NK1.1^+^) (Figure 4A,B). The results of qRT‐PCR suggested that CD300LF knockdown resulted in increased levels of pro‐inflammatory cytokines Il‐6, Il18, and decreased transcript levels of neurotrophic and anti‐inflammatory cytokines such as *Bdnf*, *Igf‐1*, *Lif*, *Il‐4*, and *Il‐10* (Figure 4C). Anatomically, CD300LF effectively reduced the lesions after TBI (Figure 4D,E). Behaviorally, it promoted motor and memory recovery as shown by mNSS scores, ORF test, rotarod test, and Y‐maze (Figure 4F–I). These results demonstrate that CD300LF protects neurological function in mice after TBI by attenuating leukocyte infiltration and inflammatory cytokine production in the TBI brain.

### 3.5 Microglia are involved in the protective effect of CD300LF on TBI

**FIGURE 5:**
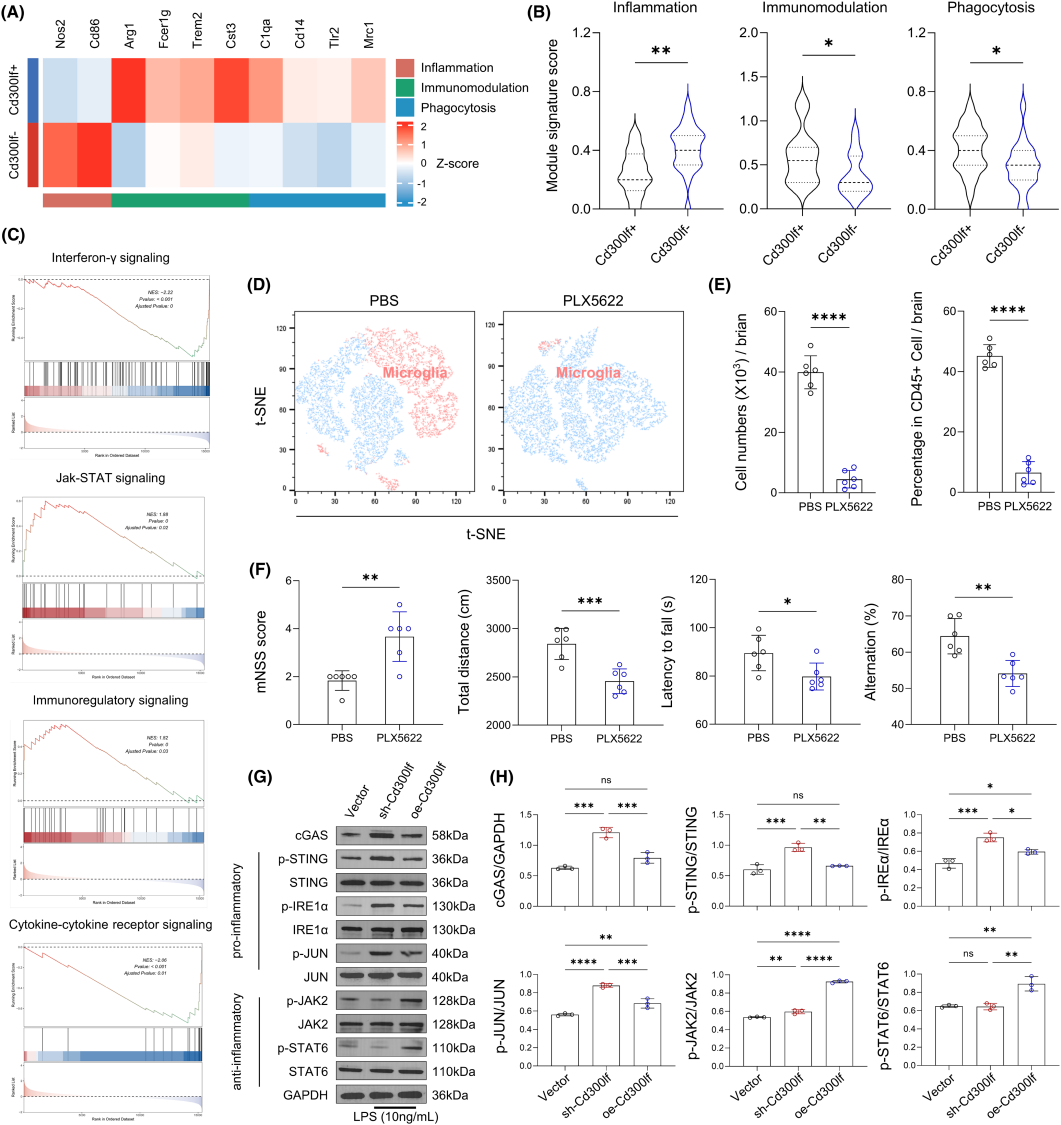
The immunomodulatory effects of CD300LF cannot be separated from the presence of microglia. (A) Heatmap of transcriptome differences between Cd300lf+ and Cd300lf− microglia. (B) The boxplots showing the distribution of the module gene set score for pro‐inflammation, anti‐inflammation, and phagocytosis among each group. (C) GSEA results of transcriptome differences between Cd300lf^+^ and Cd300lf^−^ microglia. (D, E) Flow cytometry was used to detect changes in the number of microglia after PLX5622 application and to quantify the number as well as the proportion of microglia. (F) Results of behavioral testing after using the PLX5622 (mNSS score, ORF, Rotarod test and Y‐maze.). (G, H) Representative western blots result of downstream signaling pathways and grayscale values were analyzed for relative quantification. *n* = 6/group (E, F); *n* = 3/group (H). Data are presented as mean ± SD. *p < 0.05, **p < 0.01, ***p < 0.001, ****p < 0.0001. Statistical analyses were performed using two‐tailed unpaired Student's *t* test (B, E, F) and one‐way ANOVA followed by Tukey post hoc test (H).

We first performed hierarchical clustering of the transcriptomes of CD300LF^+^ microglia and CD300LF^−^ microglia. The expression of inflammatory genes (*Nos2* and *Cd86*) was reduced in CD300LF^+^ microglia. In contrast, the expression of immunomodulatory genes (*Arg1*, *Fcer1g*, *Trem2*, and *Cst3*) and phagocytosis‐related genes (*C1qa*, *Cd14*, *Tlr2*, and *Mrc1*) was upregulated (Figure 5A). Results from Gene Ontology (GO) enrichment analysis showed that processes associated with inflammatory responses as well as neuronal apoptosis were downregulated in CD300LF^+^ microglia (Figure S4A). Consistent with these findings, gene signature module scores indicated that CD300LF^+^ microglia scored lower in the inflammatory gene set and higher in the immunomodulatory and phagocytic gene sets (Figure 5B). The results of gene set enrichment analysis (GSEA) and Kyoto Encyclopedia of Genes and Genomes (KEGG) enrichment analyses indicated that the JAK–STAT signaling pathway and the immunomodulatory signaling pathway were enriched in CD300LF^+^ microglial cells whereas the interferon‐γ signaling pathway and cytokine‐cytokine receptor signaling pathways were downregulated in CD300LF^+^ microglia (Figure 5C, Figure S4B).

Microglia survival requires signaling through the colony‐stimulating factor 1 receptor (CSF1R). To test whether microglia expressing CD300LF contribute to the recovery of TBI mice, we removed microglia by using the CSF1R inhibitor PLX5622.^19^ Flow cytometry analysis of microglia (CD11b^+^CD45^int^) showed effective microglia clearance in TBI mice treated with PLX5622 (Figure 5D,E). We found that the protective effect of CD300LF was lost in TBI mice treated with PLX5622, as evidenced by enlarged injured areas and increased brain water content in PLX5622‐treated mice (Figure 5A,B). In addition, enhanced glial cell reactions in PLX5622‐treated mice were manifested by a greater number of astrocytes and a larger astrocyte area, and the function of astrocytes was more biased towards the A1 phenotype while the transcription of genes related to the A2 phenotype was suppressed (Figure 5C–G). Consequently, enhancing neuroinflammatory responses in TBI mice after PLX5622 treatment resulted in a significant decrease in the number of neurons around the injured area (Figure 5H,I), as well as significant upregulation of apoptosis‐related genes (Figure 5J). All of the aforementioned results ultimately led to a significant decrease in neurological function in PLX5622‐treated mice (Figure 5F), suggesting that the protective effect of CD300LF on TBI cannot be separated from microglia.

### 3.6 The protective effect of CD300LF on TBI involves the STING and JAK–STAT signaling pathways

**FIGURE 6:**
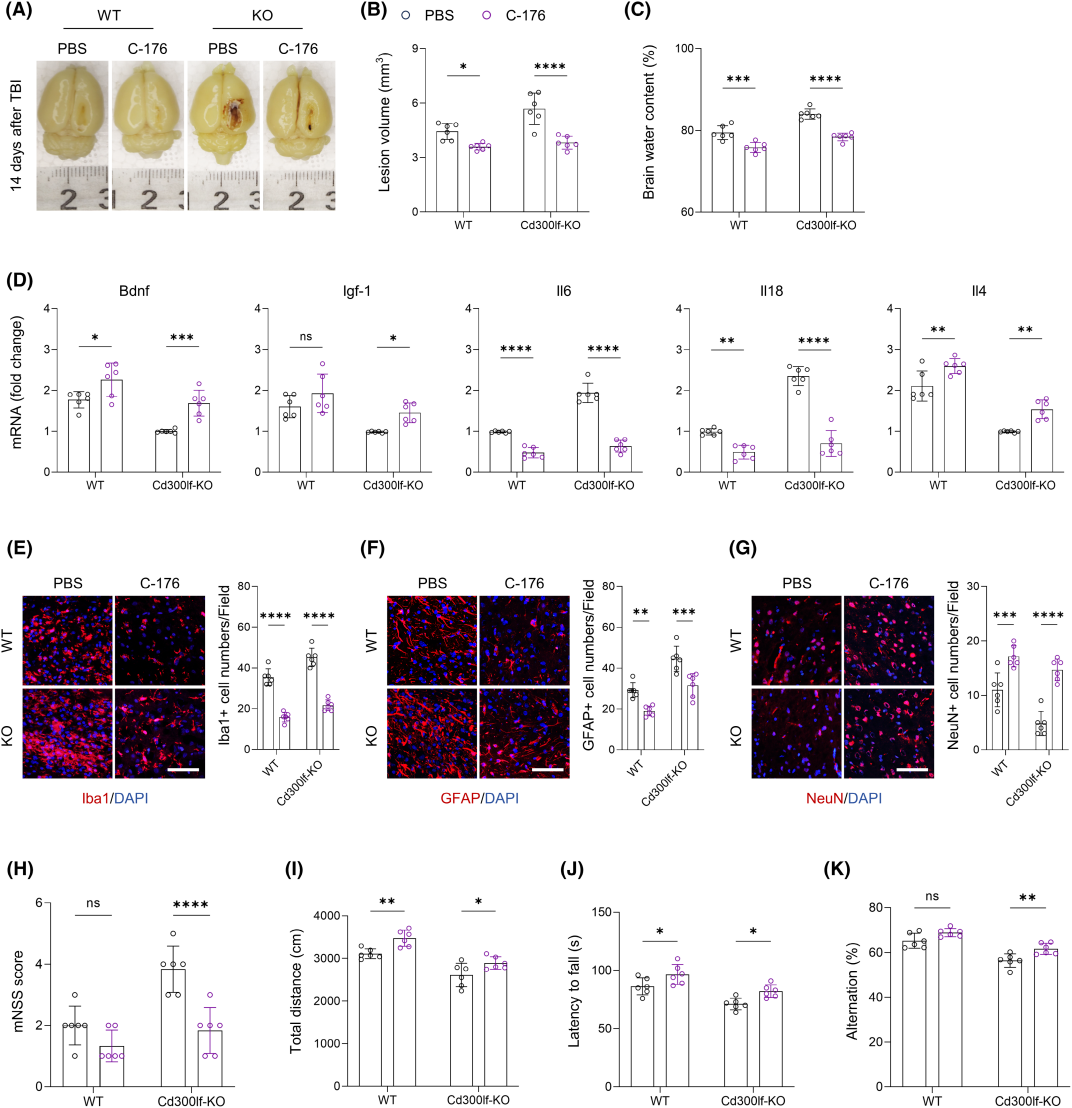
The immunomodulatory effect of CD300LF is dependent on the inhibition of the STING signaling pathway. (A–C) Representative images of the injured area in the WT and KO groups after the use of the STING pathway inhibitor C‐176 were taken and quantitatively analyzed for the volume of the injured area as well as the brain water content. (D) PCR assays were performed to detect the expression of inflammation‐associated markers in the WT and KO groups after C‐176 administration (Bdnf, Igf‐1, Il6, Il18, Il4). (E–G) Representative immunofluorescence results of microglia, astrocytes and neurons in the WT and KO groups after C‐176 administration, and quantitative analysis of cell numbers. (H–K) Behavioral test results in the WT and KO groups after C‐176 administration. H: mNSS score, I: ORF, J: Rotarod test and K: Y‐maze. *n* = 6/group. *p < 0.05, **p < 0.01, ***p < 0.001, ****p < 0.0001. Data are presented as mean ± SD. Statistical analyses were performed using two‐way ANOVA followed by Tukey post hoc test.

Considering that the STING signaling pathway is essential for interferon‐γ functionality,^20^ combined with the down‐regulation of the interferon‐γ signaling pathway in CD300LF^+^ microglia. We postulated that CD300LF may play a function in suppressing neuroinflammation after TBI by inhibiting the activity of the STING signaling pathway. The results of western‐blot suggested that the content of cGAS was significantly up‐regulated in *Cd300lf*‐knockdown microglia, along with enhanced phosphorylation levels of downstream STING, IRE1α and JUN. Some studies have reported that the enhancement of the JAK2‐STAT6 signaling pathway is closely related to the control of neuroinflammation.^21^ For this reason, we simultaneously examined the activity of the JAK–STAT signaling pathway in the sh‐*Cd300lf* microglia and oe‐*Cd300lf* microglia after LPS stimulation, and the results suggested that the phosphorylation levels of JAK2 and STAT6 were decreased in the sh‐*Cd300lf* microglia compared with those of oe‐*Cd300lf* microglia. Taken together, our results suggested that the increased activity of the STING pathway and decreased activity of the JAK2‐STAT6 pathway in KO mice may be responsible for severer neuroinflammation observed after TBI in KO mice (Figure 5G,H). Along with the changes in signaling pathways, the transcription of cytokines in microglia changed accordingly. Transcription of pro‐inflammatory cytokines (*Il6*, *Il18*) was upregulated in sh‐Cd300lf microglia; transcription of neurotrophins (*Bdnf*, *Igf1*) and inflammation‐suppressing cytokines (Il4) was upregulated in oe‐Cd300lf microglia after LPS stimulation (Figure 6A–E).

**FIGURE 7:**
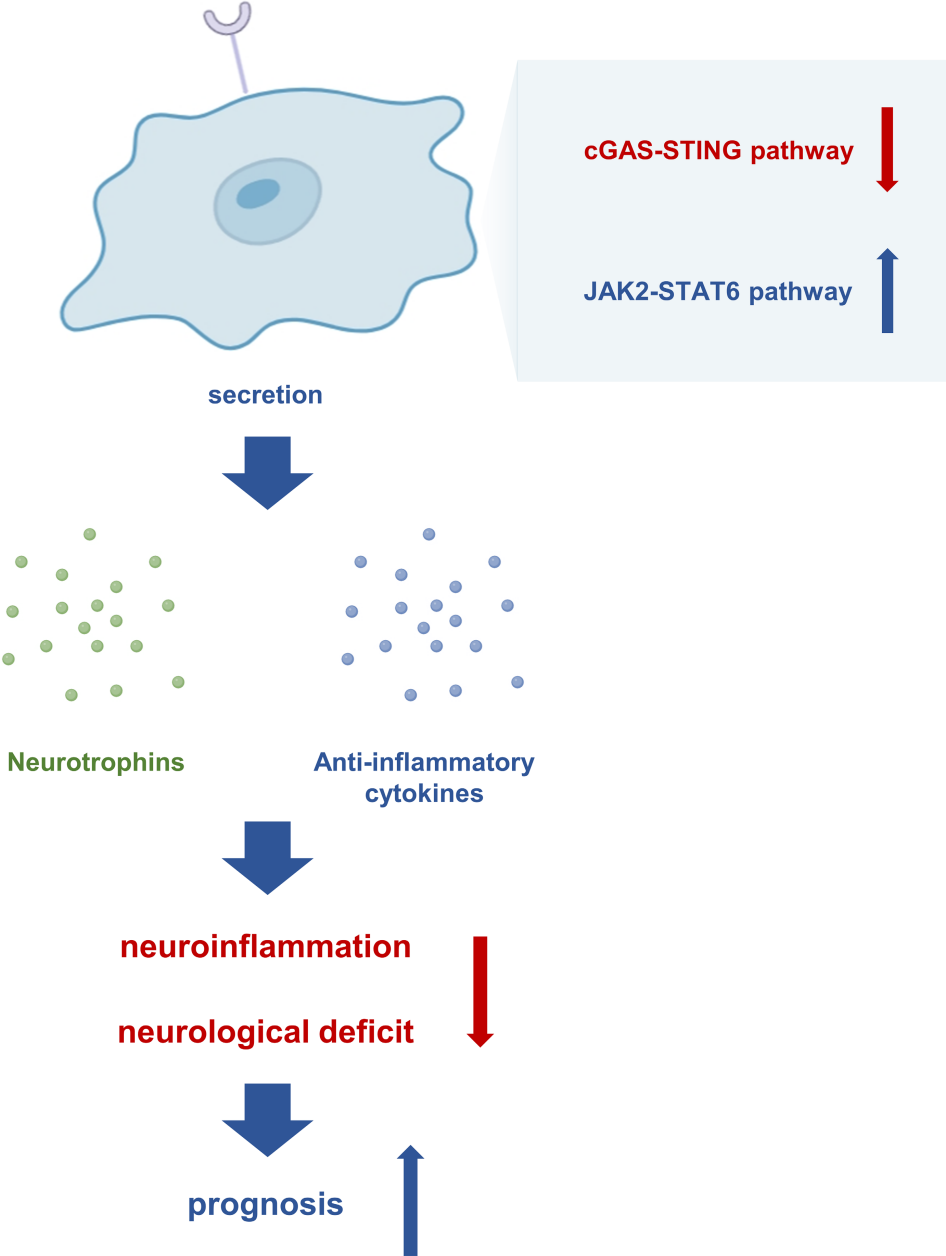
Schematic diagram of the study. CD300LF^+^ microglia inhibit neuroinflammation after TBI and promote neurological recovery in TBI patients by inhibiting the cGAS‐STING pathway while promoting JAK2‐STAT6 signaling pathway activity.

To investigate whether inhibition of STING signaling is beneficial in blocking the damaging effects caused by TBI, we applied C‐176, an inhibitor of the STING signaling pathway, on TBI mice.^22^ Notably, C‐176 treatment significantly improved the prognosis of TBI mice. The results of flow cytometry showed that the number of CD300LF^+^ microglia infiltrating around the injured area was significantly elevated in WT mice treated with C‐176 (Figure 7A,B). The increase in the number of CD300LF+ microglia led to beneficial effects. Firstly, we found that treatment with C‐176 promoted the repair of the injured area and the restoration of the blood–brain barrier function (as evidenced by a reduction in hematomas around the injured area) in TBI mice, and also reduced the level of cerebral edema in both WT mice and KO mice after TBI (Figure 6A–C).

Treatment with C‐176 also significantly reduced neuroinflammation after TBI in WT mice, especially that in KO mice. For instance, the transcript levels of pro‐inflammatory cytokines *Il6* and *Il18* were significantly decreased, while those of inflammation‐suppressing cytokines and neurotrophins *Il4*, *Bdnf*, and *Igf‐1* were increased (Figure 6D). The reactive proliferation of glial cells after TBI was also suppressed by treatment with the STING pathway inhibitor C‐176, and the number of microglia and astrocytes infiltrating around the injured area was significantly decreased, accompanied by an elevated number of neurons around the injured area (Figure 6E–G). The reduction in the level of neuroinflammation and the restoration of the number of peripheral neurons in the injured area were ultimately manifested in the improvement of neurological function in TBI mice. These results suggest that the protective effect of CD300LF on TBI involves STING and JAK–STAT pathways (Figure 6H–K).

## 4 DISCUSSION

Secondary injuries after TBI are mainly characterized by neuronal cell loss, apoptosis, and alterations in the neuroinflammatory microenvironment leading to neurological dysfunction.^3^ Recent human and animal studies increasingly support the notion that persistent neuroinflammation due to glial overactivation post‐TBI is a pivotal cause of secondary injury.^2^ In this study, we have identified, for the first time, that CD300LF‐positive microglia may attenuate neuroinflammation and protect neurons after TBI by dampening the overreaction of the residual microglia and astrocytes. Furthermore, a higher presence of CD300LF‐positive microglia in the hippocampal region post‐TBI may suggest their role in safeguarding neurological function in patients. Insights from our study on CD300LF‐positive microglia could pave the way for novel approaches in future clinical research post‐TBI.

Microglia, as the predominant immune cells in the central nervous system, play a crucial role in shaping the neuroimmune microenvironment.^23^, ^24^ It is widely recognized that pro‐inflammatory microglia are primarily activated by damage‐associated molecular patterns (DAMP), free radicals, or pro‐inflammatory cytokines. This activation results in increased production of chemokines, pro‐inflammatory cytokines, reactive oxygen species production, along with a reduction in cellular phagocytosis activity.^8^, ^25^ Excessive activation of microglia leads to the release of high levels of inflammatory factors and neurotoxic mediators, which further exacerbate neuroinflammation, leading to neuronal death and neurological dysfunction. Although pro‐inflammatory microglia are often considered harmful, it's also known that cytokines such as IL‐4 and IL‐10 can induce an anti‐inflammatory state in microglia, which is characterized by enhanced production of anti‐inflammatory cytokine and increased phagocytosis.^18^, ^26^, ^27^ However, microglia function varies with time after TBI and across TBI models, with most activated microglia displaying a mix of pro‐ and anti‐inflammatory roles. Therefore, early therapeutic interventions for TBI should aim not only to suppress microglial activation but also to modulate the microglial response to promote inflammation resolution post‐injury.^9^, ^28^, ^29^, ^30^

CD300LF was originally identified as a cellular receptor for noroviruses and has an important role in norovirus binding and entry into cells.^11^ It's role in the CNS, particularly after brain injury, has not been thoroughly examined.^12^ Leveraging single‐cell sequencing data of mouse brain tissue, we observed a notable upregulation of CD300LF in microglia following TBI. This finding was corroborated by analyzing contused brain tissue obtained intraoperatively from TBI patients. Based on these findings, we conducted an in‐depth investigation of the role of CD300LF in modulating neuroinflammation after TBI. Our findings reveal that CD300LF^+^ microglia protected neurons by inhibiting the overactivation of microglia and astrocytes after TBI, which eventually allowed neuroinflammation to be quiescent and promoted the recovery of neurological function. The underlying mechanisms involved the suppression of the overactive cGAS‐STING pathway and the restoration of the activity of the JAK2‐STAT6 signaling pathway (Figure 7). This investigation unveils, for the first time, the presence, functional dynamics, and prospective therapeutic relevance of the CD300LF‐positive microglia subpopulation post‐TBI. These insights present novel avenues and targets for mitigating neuroinflammation following TBI.

Certainly, our study has its limitations. Due to the relatively streamlined experimental design, the specific regulatory mechanisms between CD300LF and related pathways have not been explored in depth. Furthermore, how CD300LF^+^ microglia regulate the response of overactivated glial cells in the injured area through intercellular communication was not elucidated in this study either. How microglia subpopulations change in response under conditions of Cd300lf knockdown has also not been explored, data from single‐cell sequencing of WT mice versus KO mice may help answer this question. Finally, the pharmacological targeting of treatments may be problematic, and ways to improve the targeting of C‐176 are also worth exploring. Therefore, in the next step, we will further explore the relationship between CD300LF and downstream pathways, as well as the inter‐cellular regulatory mechanism, which will provide important pre‐clinical evidence for the role played by CD300LF.

## 5 CONCLUSION

In summary, combining data from single‐cell sequencing and specimens from patients with TBI, we identified the presence of a population of immunomodulatory CD300LF^+^ microglia following TBI. CD300LF^+^ microglia contribute to the suppression of neuroinflammation and promote the restoration of neurological function by inhibiting the activated STING signaling pathway and restoring the activity of the JAK2‐STAT6 signaling pathway that underwent inhibition after TBI. After PLX5622 treatment, we determined that the favorable functioning of CD300LF is not separated from the involvement of microglia. C‐176, a selective inhibitor of the STING signaling pathway, significantly increased the number and proportion of CD300LF+ microglia in the injured area and helped TBI mice to recover rapidly. This study will provide a new experimental basis for drug development and treatment by clinicians.

## AUTHOR CONTRIBUTIONS

Conceptualization: ZCL, ZHL, CXW, RJ, YY, and PPG. Methodology: ZCL, ZHL, CXW, ZHW, WW, JFC, XJZ, YL, JWZ, RJ, YY, and PPG. Validation: ZCL, ZHL, CXW, ZHW, WW, JFC, XJZ, YL, JWZ, RJ, YY, and PPG. Formal analysis: ZCL, ZHL, CXW, ZXJ, and PPG. Writing—original draft preparation: ZCL, ZHL, CXW, RJ, and PPG. Writing—review and editing: ZHW, JFC, XJZ, RJ, and YY. Visualization: ZCL and ZHL. Supervision: ZCL and PPG. All authors have read and agreed to the published version of the manuscript.

## CONFLICT OF INTEREST STATEMENT

The authors declare that they have no known competing financial interests or personal relationships that could have appeared to influence the work reported in this article.

## Supporting information


Appendix S1.

## Data Availability

The data supporting this study's findings are available from the corresponding author upon reasonable request.
