# Supplementary material for: CD300LF+ microglia impede the neuroinflammation following traumatic brain injury by inhibiting STING pathway
Source: CNS Neurosci Ther. 2024 Jul 4;30(7):e14824. doi: 10.1111/cns.14824 (PMC11224125; doi:10.1111/cns.14824)
Supplement: Supplementary file 1 — Appendix S1. [file CNS-30-e14824-s001.zip › Supplementary materials.docx]

**Supplementary materials for**

**CD300LF^+^ microglia impede the neuroinflammation following Traumatic brain injury by inhibiting STING pathway**

Zhichao Lu^1,2,3,4, #^, Zongheng Liu^5, #^, Chenxing Wang^1,2,3,4, #^, Rui Jiang^1,2,3,4^, Ziheng Wang^4,6^, Weiquan Liao^1,2,3,4^, Wei Wang^7^, Jianfeng Chen^8^, Xingjia Zhu^1,2,3,4^, Jingwei Zhao^9^, Qianqian Liu^1,2,3,4*^, Yang Yang^10*^, Peipei Gong^1,2,3,4*^

^1^ Department of Neurosurgery, Affiliated Hospital of Nantong University, Medical School of Nantong University, Nantong, Jiangsu, 226001, China.

^2^ Neuro-Microscopy and Minimally Invasive Translational Medicine Innovation Center, Affiliated Hospital of Nantong University, Nantong, Jiangsu, 226001, China.

^3^ Jiangsu Medical Innovation Centre, Neurological disease diagnosis and treatment center, Affiliated Hospital of Nantong University, Nantong, Jiangsu, 226001, China.

^4^ Research Center of Clinical Medicine, Affiliated Hospital of Nantong University, Nantong, Jiangsu, 226001, China.

^5^ Department of Neurosurgery, Zhejiang Provincial Hospital of Chinese Medicine, The First Affiliated Hospital of Zhejiang Chinese Medical University, Hangzhou, 310060, China.

^6^ Department of Biobank, Affiliated Hospital of Nantong University, Nantong, Jiangsu, 226001, China.

^7^ Department of Pathology, Affiliated Hospital of Nantong University, Affiliated Hospital of Nantong University, Nantong, Jiangsu, 226001, China.

^8^ Department of Orthopedics and Traumatology, Wuxi TCM Hospital Affiliated to Nanjing University of Chinese Medicine, Wuxi, Jiangsu, 214071, China.

^9^ Department of General Surgery, Xinhua Hospital Affiliated to Shanghai Jiao Tong University School of Medicine, Shanghai Key Laboratory of Biliary Tract Disease Research, Research Institute of Biliary Tract Disease, Shanghai, 200092, China.

^10^ Department of Neurosurgery, Southwest Hospital, Third Military Medical University (Army Medical University), Chongqing, 400038, China.

^11^ Lead contact.

# These authors contributed equally

* Corresponding authors

Peipei Gong: ntgpp@ntu.edu.cn

Yang Yang: yangyang200905@tmmu.edu.cn

Qianqian Liu: ntfyliuqianqian@163.com

**Supplementary Figures**

**
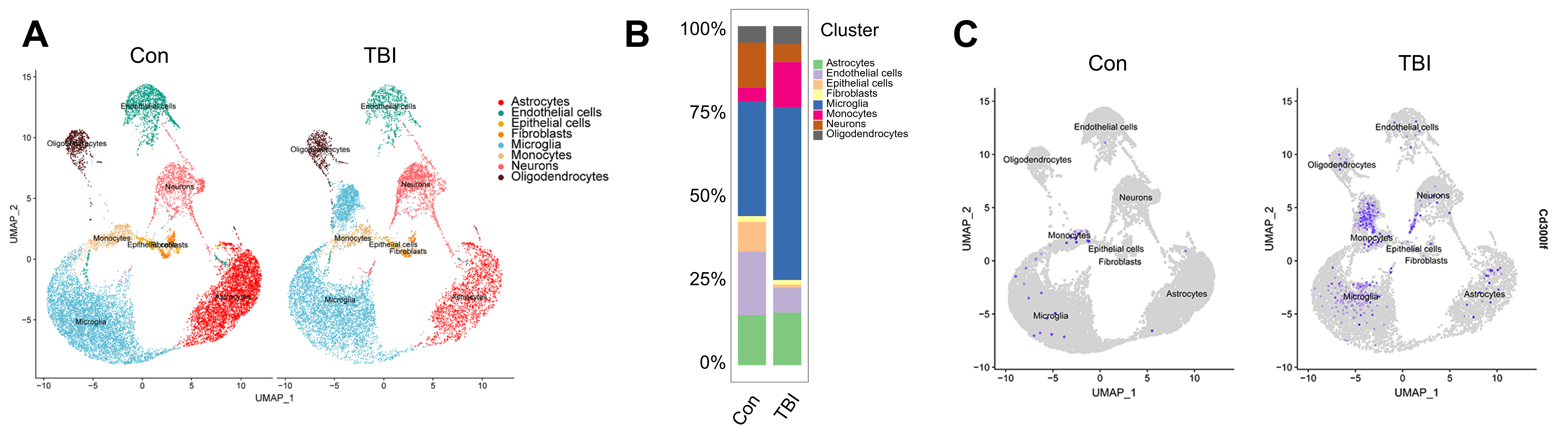
**

**Supplementary Figure. 1 Cd300lf expression is upregulated in microglia after TBI.** **(A)** Representative UMAP results of single-cell sequencing data of brain tissue before and after TBI. **(B)** Quantitative analysis was performed for the percentage of each cell type before and after TBI. **(C)** Representative UMAP results of CD300lf expression before and after TBI, and the cells expressing CD300lf are labeled in purple.


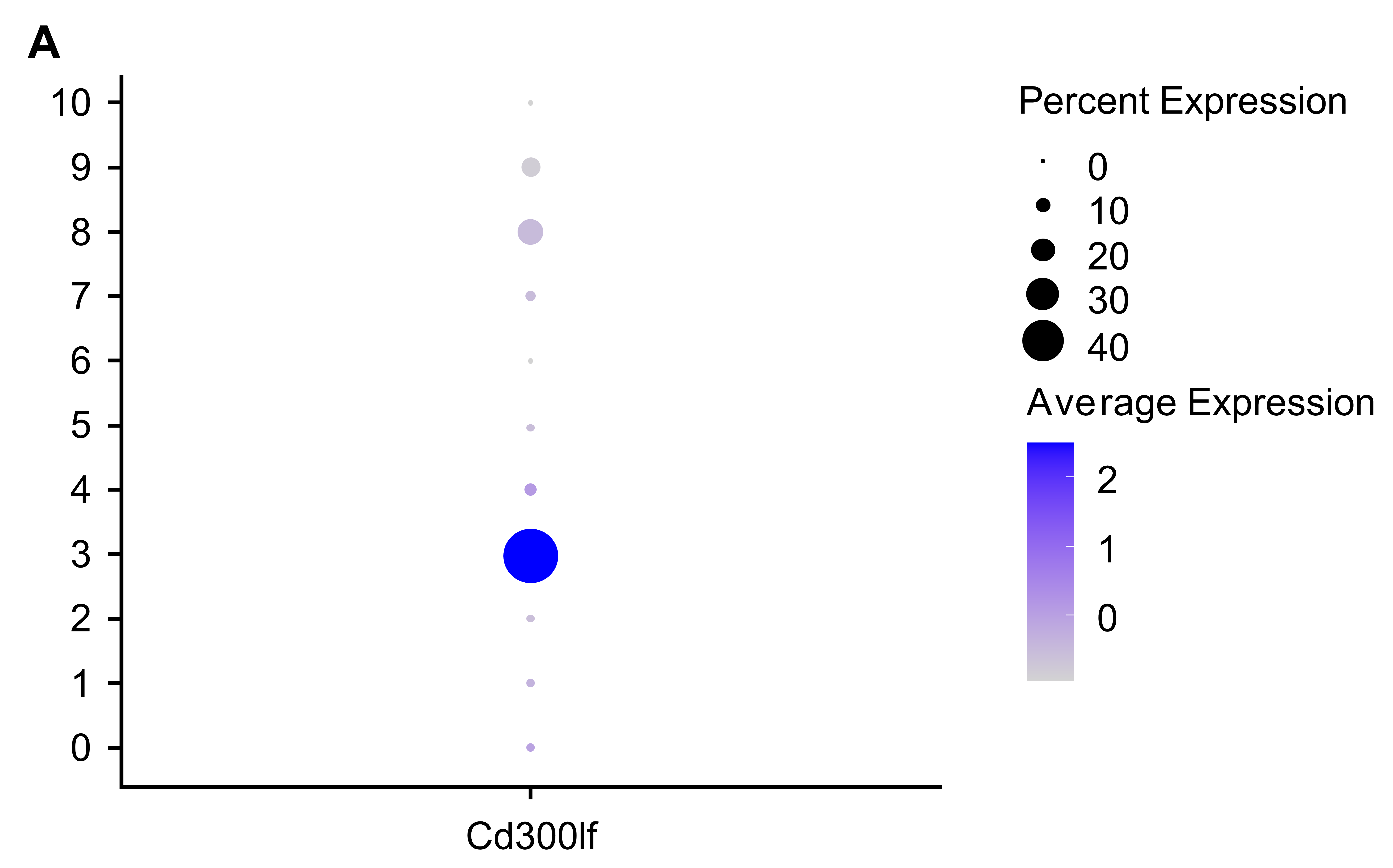


**Supplementary Figure. 2 Cd300lf is mainly expressed in cluster 3 microglia among other clusters after TBI. (A)** Single-cell sequencing of brain cells from TBI mice. Dot plots showing that *Cd300lf* was mainly expressed in cluster 3 microglia after TBI.


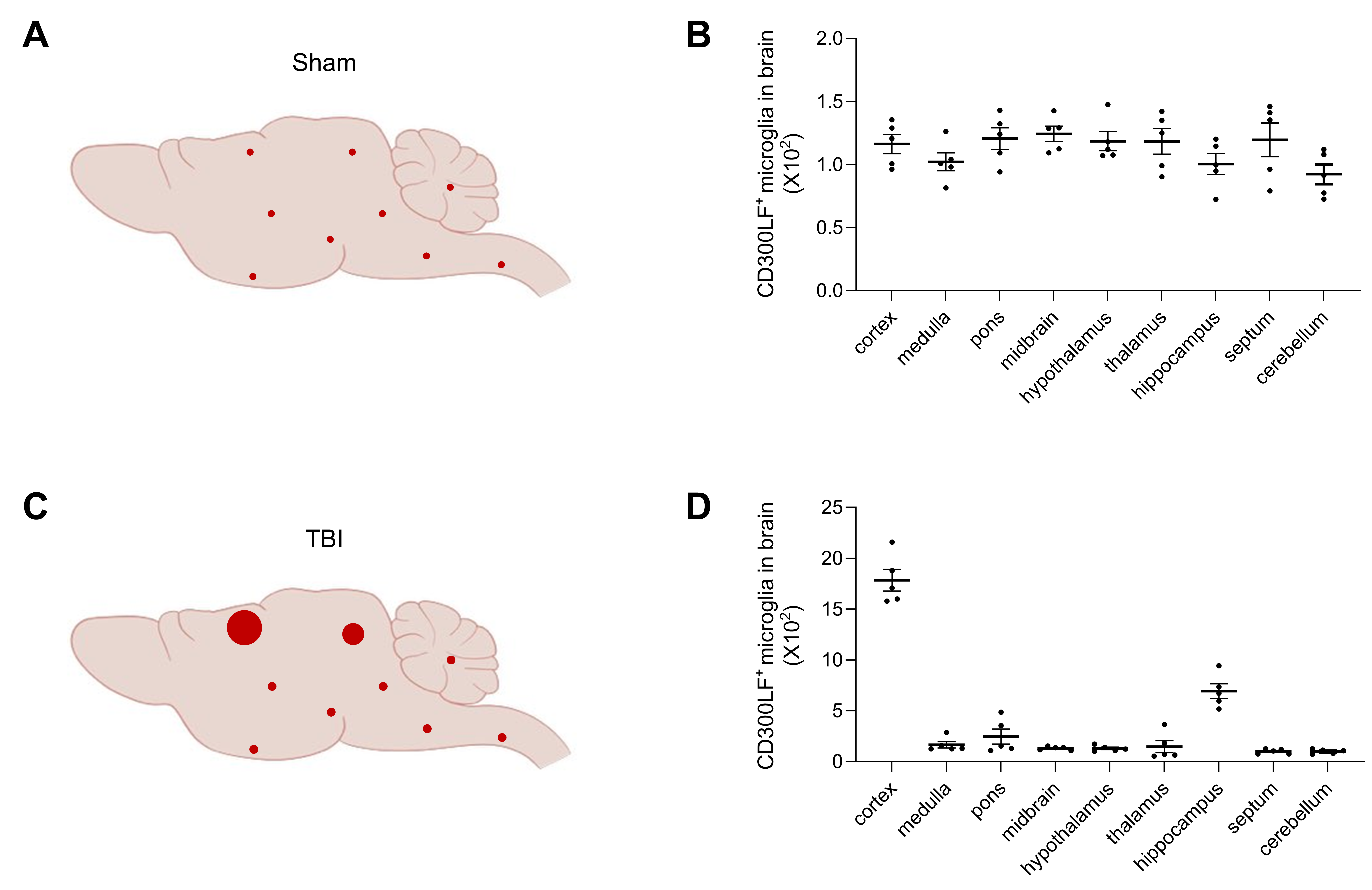


**Supplementary Figure. 3 CD300LF^+^ microglia aggregated in the injured area cortex and hippocampus. (A-B)** Schematic representation of the uniform distribution of CD300LF^+^ microglia in normal mouse brain, then quantitative statistics. **(C-D)** Schematic representation of CD300LF^+^ microglia aggregated in the injured area cortex and hippocampus, then quantitative statistics. n=5/group. Data are presented as mean ± SEM.


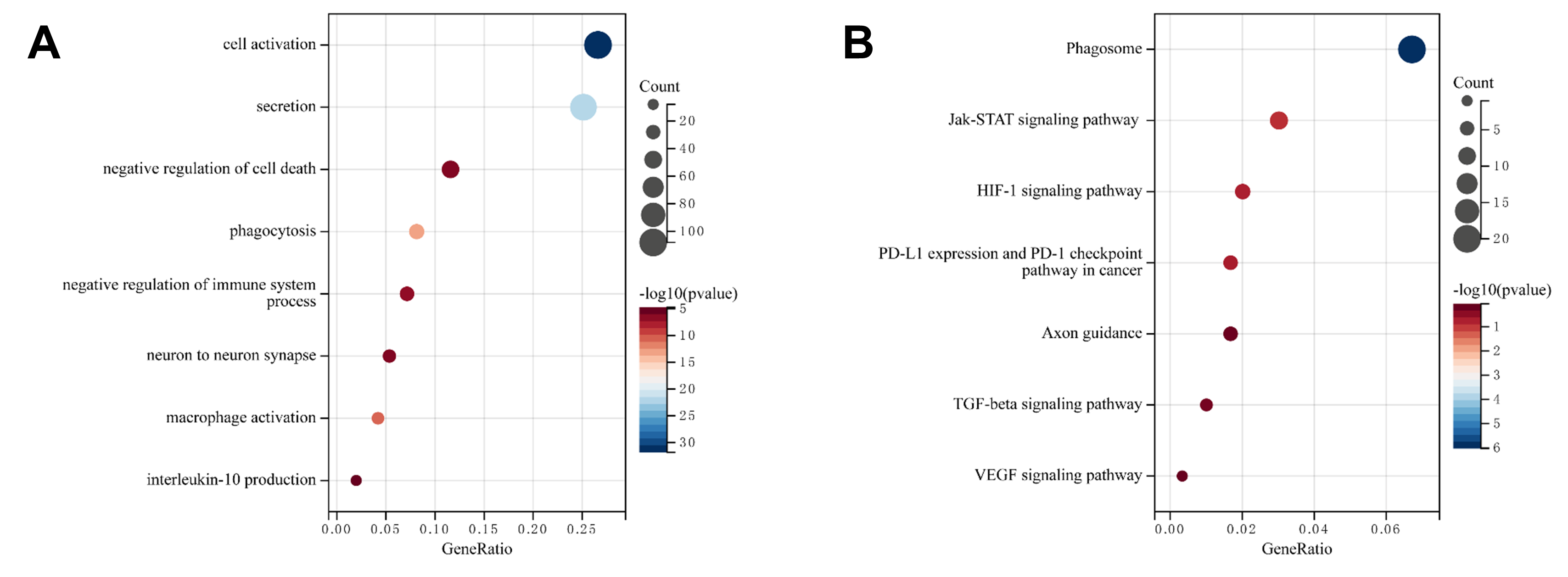


**Supplementary Figure. 4 Enrichment analysis results from single-cell sequencing. (A-B)** Results of KEGG and GO enrichment analysis of Cd300lf^+^ microglia compared to Cd300lf^-^ microglia.


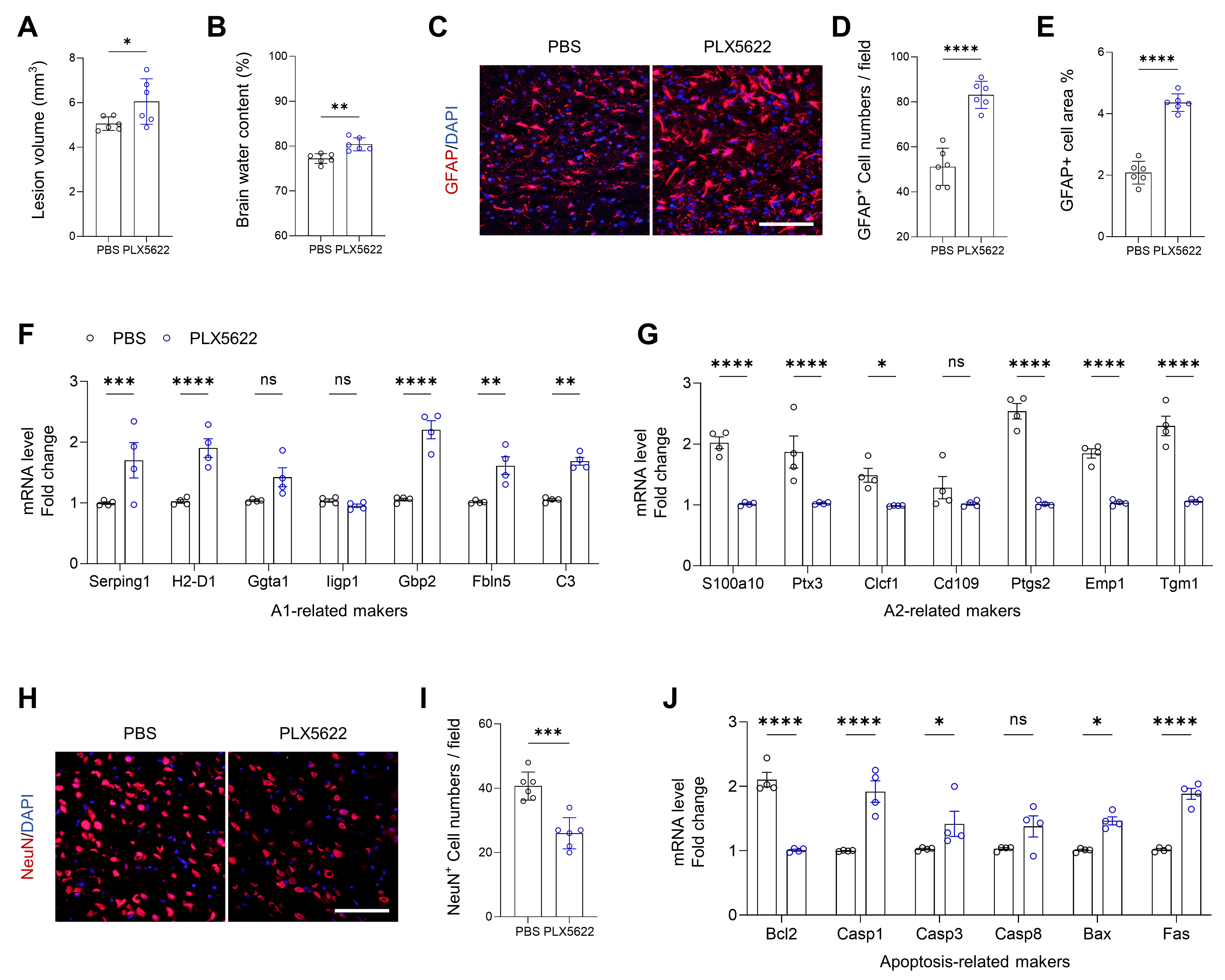


**Supplementary Figure. 5 The immunomodulatory effects of CD300LF cannot be separated from the presence of microglia. (A-B)** Quantitative statistical results of the area of injured area as well as water content before and after the use of PLX5622. **(C-E)** Representative astrocyte immunofluorescence results before and after PLX5622 administration, with quantitative analysis of cell numbers and GFAP+ area region. **(F-G)** PCR assays were performed to detect the expression of astrocyte activation markers before and after the use of PLX5622, and relative quantitative analysis was performed. **(H-I)** Representative neuron immunofluorescence results before and after PLX5622 administration, with quantitative analysis of cell numbers. **(J)** PCR assays were performed to detect the expression of apoptosis-related markers before and after the use of PLX5622, and relative quantitative analysis was performed. n=6/group. Data are presented as mean ± SD. *P < 0.05, **P < 0.01, ***P < 0.001, ****P < 0.0001. Statistical analyses were performed using two-tailed unpaired Student’s t test.


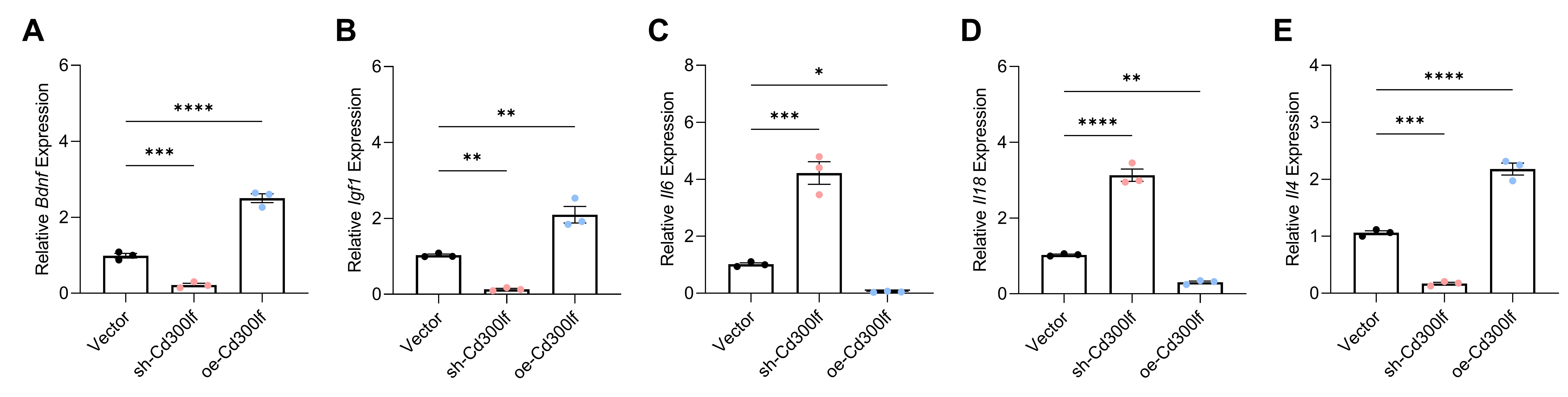


**Supplementary Figure. 6 Cd300lf modulates LPS-induced microglia inflammation in vitro. (A-D)** PCR assays were performed to detect the expression of *Bdnf*, *Igf-1*, *Il6*, *Il18* and *Il4* in Vector group, sh-Cd300lf group and oe-Cd300lf group after LPS (10ng/mL) stimulation. Relative quantitative analysis was performed. Data are presented as mean ± SD. *P < 0.05, **P < 0.01, ***P < 0.001, ****P < 0.0001. Statistical analyses were performed using one-way ANOVA followed by Tukey post hoc test.


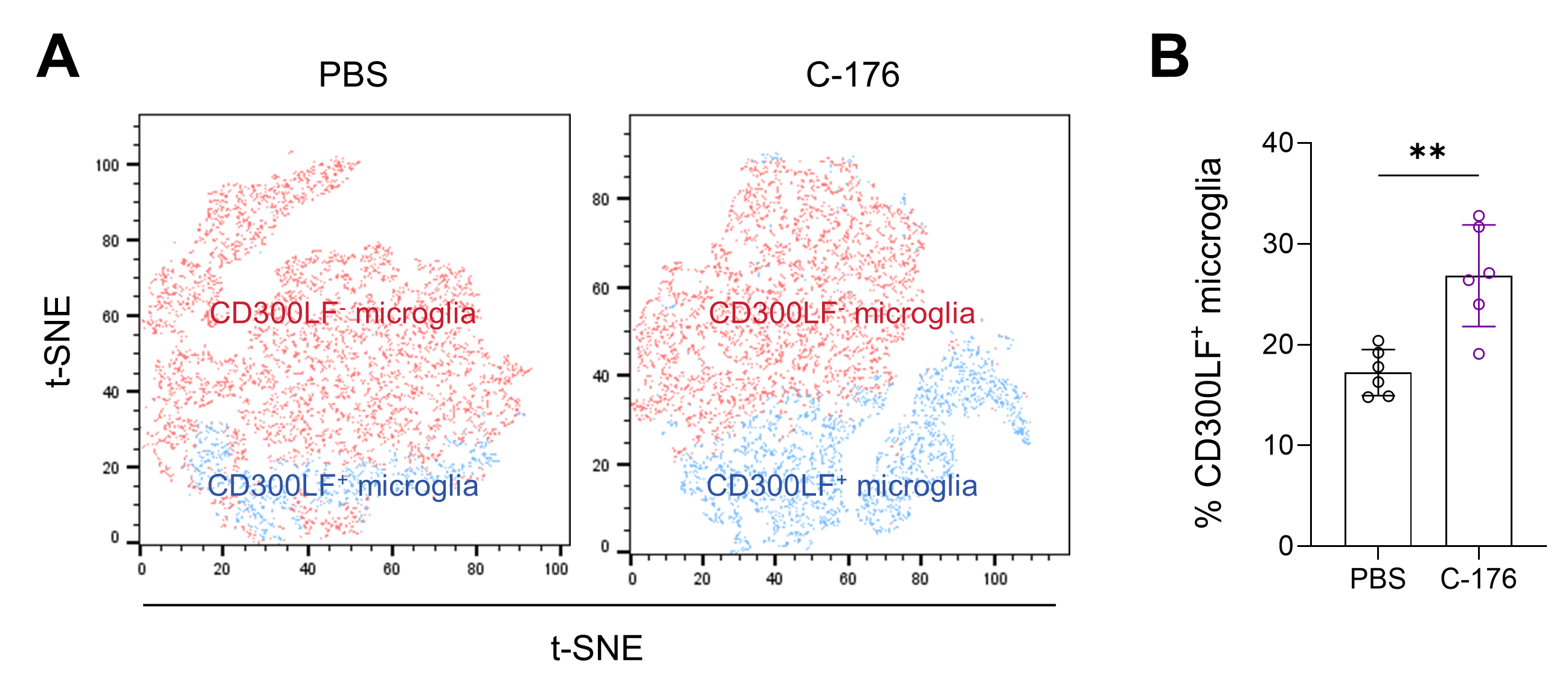


**Supplementary Figure. 7 C-176 increased the population of CD300LF^+^ microglia. (A-B)** Representative t-SNE results of CD300LF+ microglia before and after C-176 treatment and quantitative analysis of the proportion of CD300LF+ microglia to all microglia. n=6/group. Data are presented as mean ± SD. **P < 0.01. Statistical analyses were performed using two-tailed unpaired Student’s t test.

**Supplementary Tables**

**Supplementary Table. 1 Basic Clinical Information for TBI Patients.**

| **Patient number** | **1** | **2** | **3** | **4** | **5** | **6** |
| --- | --- | --- | --- | --- | --- | --- |
| Age (year) | 58 | 52 | 55 | 63 | 52 | 64 |
| Sex (male) | M | F | M | F | M | F |
| GCS before operative | 8 | 7 | 10 | 6 | 9 | 8 |
| Length of hospitalization (days) | 11 | 12 | 9 | 14 | 13 | 12 |
| GCS at discharge | 12 | 11 | 13 | 9 | 10 | 12 |

**Supplementary Table. 2 PCR sequences.**

| **Gene** | **Forward primer sequence (5’-3’)** | **Reverse primer sequence (5’-3’)** |
| --- | --- | --- |
| *Cd300lf* (ms) | GATGCTGGCATTTACTGGTGTGG | GGTTGTCACTGTGAAGATGGTGG |
| *CD300LF* (hu) | TTTCCTCTGCCCAGGTTGACCA | CCATGTTGCAGTAGGTCGGTTC |
| *Cd86* | ACGTATTGGAAGGAGATTACAGCT | TCTGTCAGCGTTACTATCCCGC |
| *Nos2* | GAGACAGGGAAGTCTGAAGCAC | CCAGCAGTAGTTGCTCCTCTTC |
| *Mrc1* | GTTCACCTGGAGTGATGGTTCTC | AGGACATGCCAGGGTCACCTTT |
| *Vegfa* | CTGCTGTAACGATGAAGCCCTG | GCTGTAGGAAGCTCATCTCTCC |
| *Arg1* | TCATCTGGGTGGATGCTCACAC | GAGAATCCTGGCACATCGGGAA |
| *Pdgfa* | GCAGTTGCCTTACGACTCCAGA | GGTTTGAGCATCTTCACAGCCAC |
| *Serping1* | TTGCCTGTGTCCACCAAGCACT | GCTGCTTCCATACAGGCTCTGA |
| *H2-D1* | TGAGGAACCTGCTCGGCTACTA | GGTCTTCGTTCAGGGCGATGTA |
| *Ggta1* | GCTGATTGTCTCAACCGTGGTTG | CTGCCATCTGTTCTCACCAACC |
| *Iigp1* | GGACAAGCTGATAAGTGACCTCC | CCAGCCAAATCCTCTGCTTCAG |
| *Gbp2* | ACATGCCCACAGAAACCCTCCA | AGGCATCTCGTTTGGCTTCCAG |
| *Fbln5* | CCTGTGTCAACACCTATGGCTC | CACACTCGTGTTGACAGAGGAAC |
| *C3* | CGCAACGAACAGGTGGAGATCA | CTGGAAGTAGCGATTCTTGGCG |
| *S100a10* | AACAAAGGAGGACCTGAGAGTAC | CTTTGCCATCTCTACACTGGTCC |
| *Ptx3* | CGAAATAGACAATGGACTTCATCC | CATCTGCGAGTTCTCCAGCATG |
| *Clcf1* | GGAGCATCAACTCCGCAGCTTA | CCACACTTCCAAGTTGACCGTG |
| *Cd109* | CATGGATTTGGCTAGACGCCTAC | GCTCTGCTGGAACGGTTGTTAAC |
| *Ptgs2* | GCGACATACTCAAGCAGGAGCA | AGTGGTAACCGCTCAGGTGTTG |
| *Emp1* | TCCCTGTCCTACGGCAATGAAG | CTGGAACACGAAGACCACAAGG |
| *Tgm1* | ATCTGCCCTCAGGCTTTGATGG | CGTTCTTGACGGACTCCACAGA |
| *Bcl2* | CCTGTGGATGACTGAGTACCTG | AGCCAGGAGAAATCAAACAGAGG |
| *Casp1* | GGCACATTTCCAGGACTGACTG | GCAAGACGTGTACGAGTGGTTG |
| *Casp3* | GGAGTCTGACTGGAAAGCCGAA | CTTCTGGCAAGCCATCTCCTCA |
| *Casp8* | ATGGCTACGGTGAAGAACTGCG | TAGTTCACGCCAGTCAGGATGC |
| *Bax* | AGGATGCGTCCACCAAGAAGCT | TCCGTGTCCACGTCAGCAATCA |
| *Fas* | CTGCGATTCTCCTGGCTGTGAA | CAACAACCATAGGCGATTTCTGG |
| *Nestin* | AGGAGAAGCAGGGTCTACAGAG | AGTTCTCAGCCTCCAGCAGAGT |
| *Map2* | GCTGTAGCAGTCCTGAAAGGTG | CTTCCTCCACTGTGGCTGTTTG |
| *P16* | TGTTGAGGCTAGAGAGGATCTTG | CGAATCTGCACCGTAGTTGAGC |
| *P21* | TCGCTGTCTTGCACTCTGGTGT | CCAATCTGCGCTTGGAGTGATAG |
| *Bdnf* | GGCTGACACTTTTGAGCACGTC | CTCCAAAGGCACTTGACTGCTG |
| *Igf-1* | GTGGATGCTCTTCAGTTCGTGTG | TCCAGTCTCCTCAGATCACAGC |
| *Lif* | TCAACTGGCACAGCTCAATGGC | GGAAGTCTGTCATGTTAGGCGC |
| *Il6* | TACCACTTCACAAGTCGGAGGC | CTGCAAGTGCATCATCGTTGTTC |
| *Il18* | GACAGCCTGTGTTCGAGGATATG | TGTTCTTACAGGAGAGGGTAGAC |
| *Il4* | ATCATCGGCATTTTGAACGAGGTC | ACCTTGGAAGCCCTACAGACGA |
| *Il10* | CGGGAAGACAATAACTGCACCC | CGGTTAGCAGTATGTTGTCCAGC |

**Supplementary Table. 3 Antibodies used in this paper.**

|  | **Antibodies** | **Cat.#** | **Supplier** | **Dilution** | **Applications** |
| --- | --- | --- | --- | --- | --- |
| 1 | CD300LF Polyclonal antibody (13334-1-AP) | 13334-1-AP | proteintech | 1:1000 | WB |
| 2 | GFAP Monoclonal Antibody (S206A-8), FITC | MA5-45632 | Invitrogen | 1:500 | FC |
| 3 | NeuN (D4G4O) XP ® Rabbit mAb (Alexa Fluor ® 594 Conjugate) | 90171S | CST | 1:500 | FC |
| 4 | CD45 Monoclonal Antibody (HI30), Brilliant Violet™ 421, eBioscience™ | 404-0459-42 | Invitrogen | 1:500 | FC |
| 5 | CD11b (activation epitope) Monoclonal Antibody (CBRM1/5), Alexa Fluor™ 700, eBioscience™ | 16-0113-82 | Invitrogen | 1:500 | FC |
| 6 | CD300f (IREM-1) Monoclonal Antibody (UP-D1), eFluor™ 660, eBioscience™ | 50-3008-42 | Invitrogen | 1:500 | FC |
| 7 | CD3e Monoclonal Antibody (145-2C11), Brilliant Violet™ 650, eBioscience™ | 414-0031-82 | Invitrogen | 1:500 | FC |
| 8 | CD11b Monoclonal Antibody (M1/70), Super Bright™ 702, eBioscience™ | 63-0112-82 | Invitrogen | 1:500 | FC |
| 9 | NK1.1 Monoclonal Antibody (PK136), PE-Cyanine7, eBioscience™ | 25-5941-81 | Invitrogen | 1:500 | FC |
| 10 | CD19 Monoclonal Antibody (eBio1D3 (1D3)), PE-Cyanine5, eBioscience™ | 14-0193-82 | Invitrogen | 1:500 | FC |
| 11 | CD4 Monoclonal Antibody (RM4-5), Super Bright™ 600, eBioscience™ | 63-0042-82 | Invitrogen | 1:500 | FC |
| 12 | CD8a Monoclonal Antibody (53-6.7), Alexa Fluor™ 700, eBioscience™ | 56-0081-82 | Invitrogen | 1:500 | FC |
| 13 | CD45 Monoclonal Antibody (HI30), PerCP-Cyanine5.5, eBioscience™ | 45-0459-42 | Invitrogen | 1:500 | FC |
| 14 | Ly-6G Monoclonal Antibody (1A8-Ly6g), APC, eBioscience™ | 17-9668-82 | Invitrogen | 1:500 | FC |
| 15 | F4/80 Monoclonal Antibody (BM8), Brilliant Violet™ 421, eBioscience™ | 404-4801-82 | Invitrogen | 1:500 | FC |
| 16 | cGAS Recombinant Rabbit Monoclonal Antibody (10H1L5) | 703149 | Invitrogen | 1:1000 | WB |
| 17 | STING Polyclonal Antibody | PA5-20782 | Invitrogen | 1:1000 | WB |
| 18 | Phospho-STING (Ser366) Polyclonal Antibody | PA5-105674 | Invitrogen | 1:1000 | WB |
| 19 | IRE1 alpha Monoclonal Antibody (J.607.10) | MA5-14991 | Invitrogen | 1:1000 | WB |
| 20 | Phospho-IRE1 alpha (Ser724) Polyclonal Antibody | PA1-16927 | Invitrogen | 1:1000 | WB |
| 21 | c-Jun Monoclonal Antibody (5B1) | MA5-15881 | Invitrogen | 1:1000 | WB |
| 22 | Phospho-c-Jun (Ser63) Monoclonal Antibody (J.973.7) | MA5-15115 | Invitrogen | 1:1000 | WB |
| 23 | JAK2 Monoclonal Antibody (691R5) | AHO1352 | Invitrogen | 1:1000 | WB |
| 24 | Phospho-JAK2 (Tyr1007, Tyr1008) Polyclonal Antibody | 44-426G | Invitrogen | 1:1000 | WB |
| 25 | STAT6 Monoclonal Antibody (7D3) | MA5-15659 | Invitrogen | 1:1000 | WB |
| 26 | Phospho-STAT6 (Tyr641) Recombinant Rabbit Monoclonal Antibody (46H1L12) | 700247 | Invitrogen | 1:1000 | WB |
| 27 | GAPDH Monoclonal Antibody (1D4) | MA1-16757 | Invitrogen | 1:5000 | WB |
| 28 | Anti Iba1, Rabbit (for Immunocytochemistry, 019-19741) | 019-19741 | Wako | 1:500 | IF |
| 29 | GFAP Monoclonal Antibody (GA5), Alexa Fluor™ 488, eBioscience™ | 53-9892-82 | Invitrogen | 1:500 | IF |
| 30 | NeuN Recombinant Rabbit Monoclonal Antibody (14H6L24) | 702022 | Invitrogen | 1:500 | IF |
| 31 | Anti-CD86 [GL-1] (ab119857) | ab119857 | Abcam | 1:500 | IF |
| 32 | CD206 Monoclonal antibody | 12-2069-42 | proteintech | 1:500 | IF |
| 33 | Goat Anti-Mouse IgG H&L (Alexa Fluor® 488) | A28175 | Abcam | 1:1000 | IF |
| 34 | Goat Anti-Rat IgG H&L (Alexa Fluor® 488) | ab150077 | Abcam | 1:1000 | IF |
| 35 | Goat Anti-Rabbit IgG H&L (Alexa Fluor® 594) | ab150080 | Abcam | 1:1000 | IF |
| 36 | Goat Anti-Mouse IgG H&L (Alexa Fluor® 594) | ab150116 | Abcam | 1:1000 | IF |
| 37 | Goat Anti-Rabbit IgG H&L (HRP) | ab6721 | Abcam | 1:2000 | WB |
| 38 | Rabbit Anti-Mouse IgG H&L (HRP) | ab6728 | Abcam | 1:2000 | WB |

**Supplementary Table. 4 DEGs of Cluster 3 microglia.**

| **gene** | **log2FC** | **pvalue** |
| --- | --- | --- |
| Ctsd | 1.985252 | 1.10E-13 |
| Plp1 | 1.923094 | 1.13E-174 |
| C1qb | 1.903443 | 6.41E-17 |
| Hexb | 1.901599 | 5.63E-17 |
| Ptgds | 1.871707 | 3.91E-05 |
| C1qa | 1.697763 | 6.69E-19 |
| Lgmn | 1.673613 | 1.10E-18 |
| Fcrls | 1.628167 | 2.18E-16 |
| Sparc | 1.59013 | 2.73E-13 |
| Cd300lf | 1.553282 | 2.05E-156 |
| Arg1 | 1.525172 | 1.00E-11 |
| Trem2 | 1.4931 | 2.77E-14 |
| Ifi27l2a | 1.455023 | 9.13E-199 |
| Ifitm3 | 1.341439 | 6.27E-173 |
| Rgs10 | 1.302411 | 1.40E-14 |
| Gpr34 | 1.297891 | 1.92E-10 |
| Ccl5 | 1.289307 | 2.86E-123 |
| Il4 | 1.2617 | 5.90E-10 |
| Ccl12 | 1.259961 | 9.05E-63 |
| Timp2 | 1.255212 | 2.03E-08 |
| Ctss | 1.241441 | 1.30E-21 |
| Bdnf | 1.225134 | 0.152691079 |
| Vegfa | 1.104251 | 8.32E-06 |
| Il10 | 1.073816 | 0.000666726 |
| Tuba1a | -0.88415 | 3.99E-11 |
| Cebpb | -0.88631 | 0.001284591 |
| Atf3 | -0.89438 | 0.02241159 |
| Fos | -0.90595 | 0.001339642 |
| Nfkbiz | -0.90861 | 0.0008144 |
| Cxcl14 | -1.07909 | 1.06E-05 |
| Il18 | -1.10812 | 4.87E-06 |
| Il6 | -1.19849 | 6.31E-08 |
| Il1b | -1.66942 | 0.02395746 |
