# Supplementary figures and images for: CD300LF+ microglia impede the neuroinflammation following traumatic brain injury by inhibiting STING pathway
Source: CNS Neurosci Ther. 2024 Jul 4;30(7):e14824. doi: 10.1111/cns.14824 (PMC11224125; doi:10.1111/cns.14824)

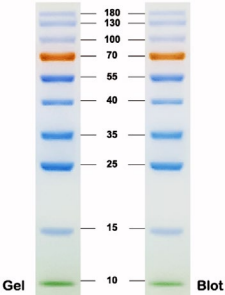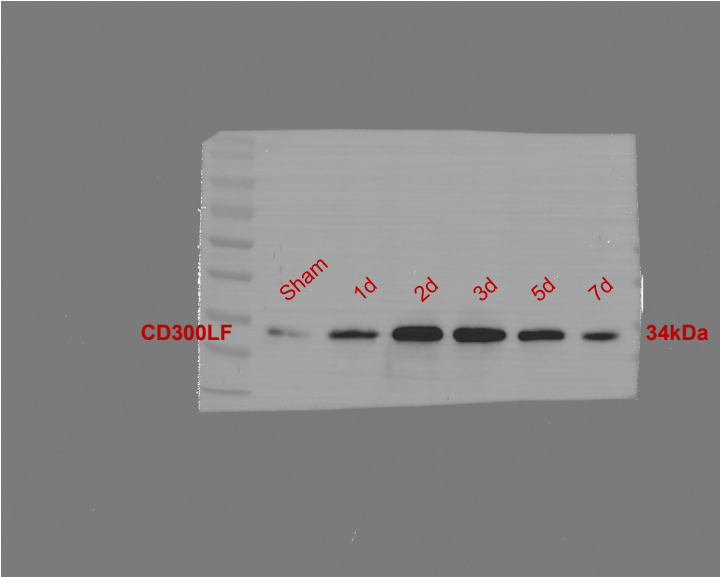

Fig 1E GAPDH

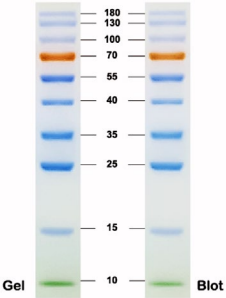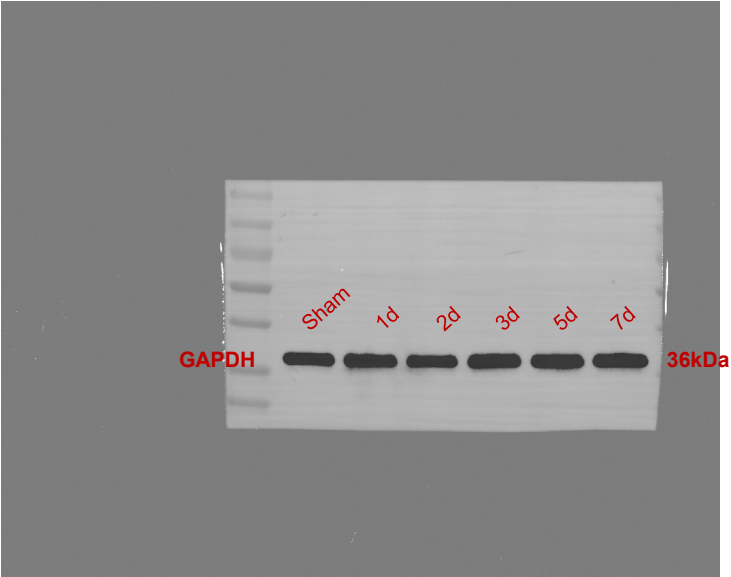

Fig 5G cGAS

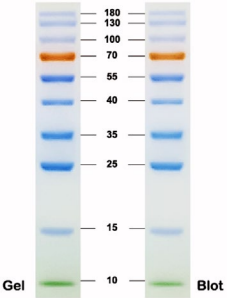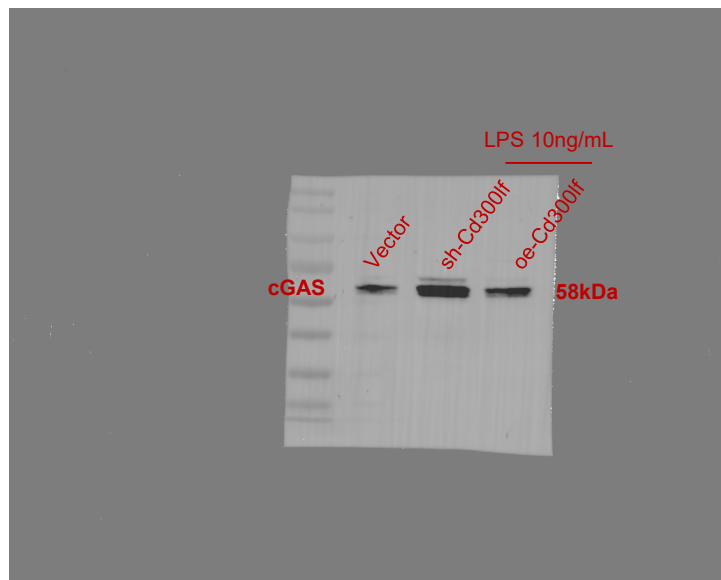

Fig 5G p-STING

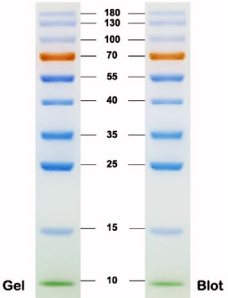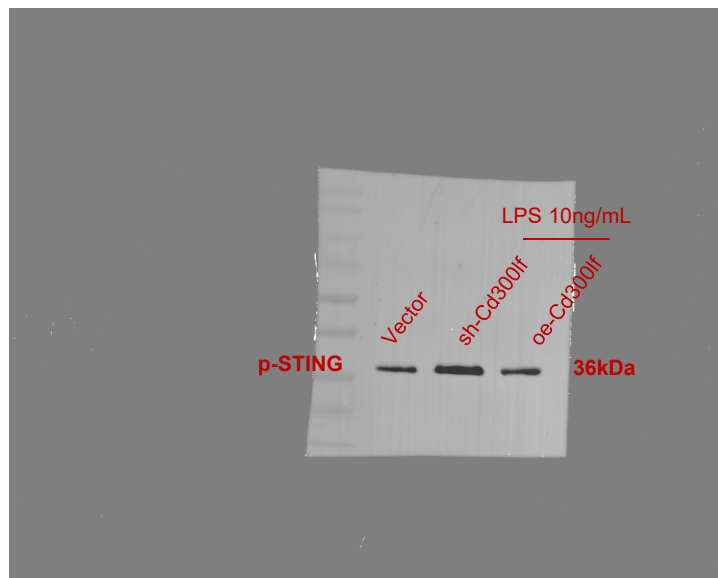

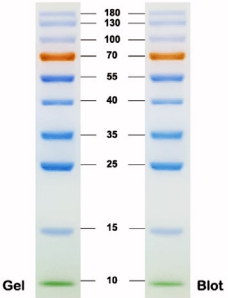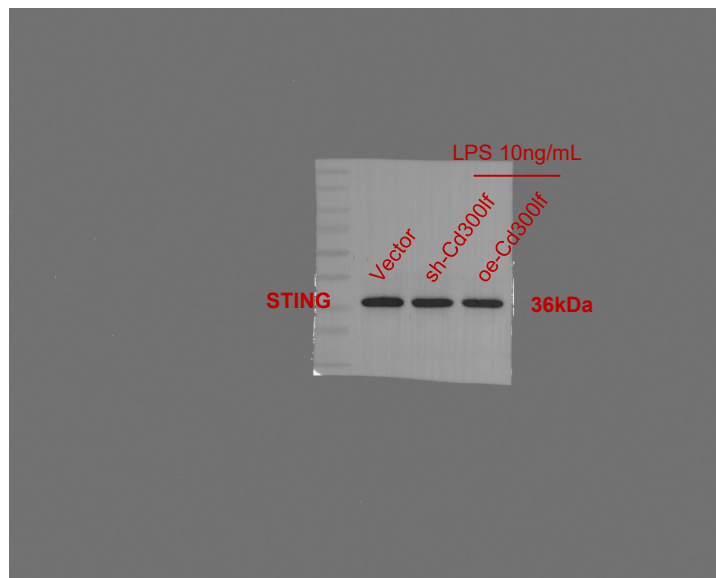

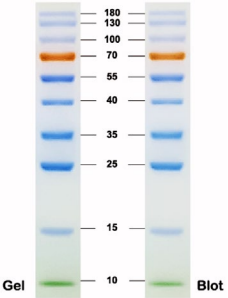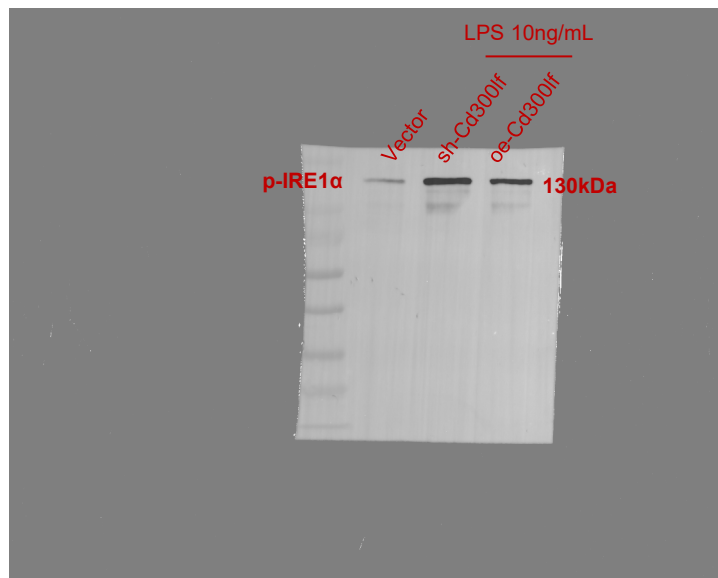

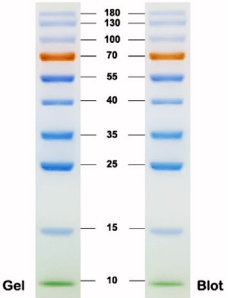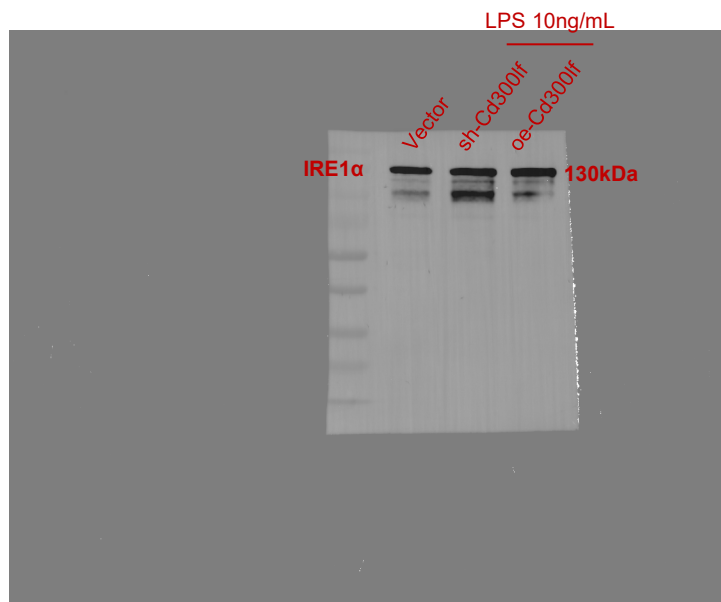

Fig 5G p-JUN

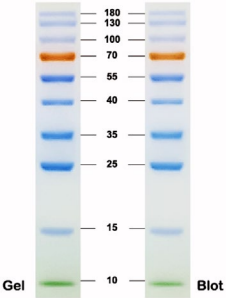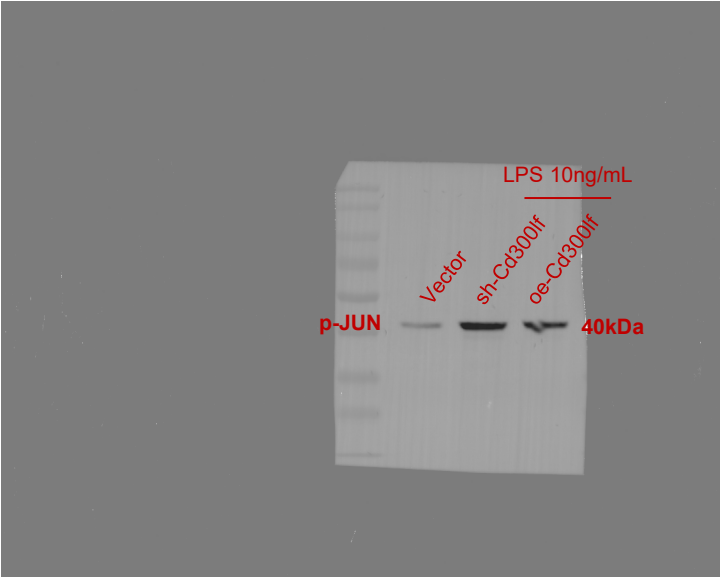

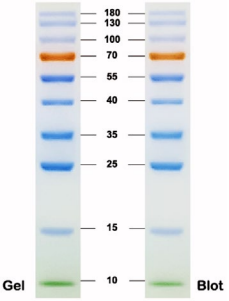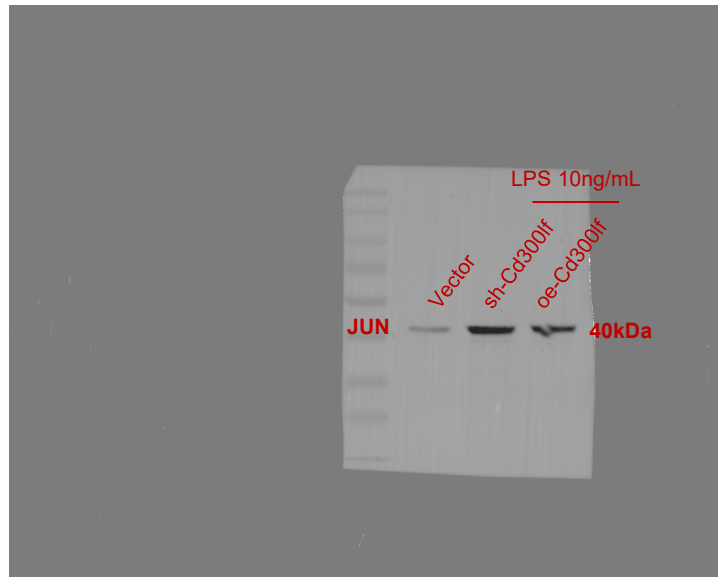

Fig 5G p-JAK2

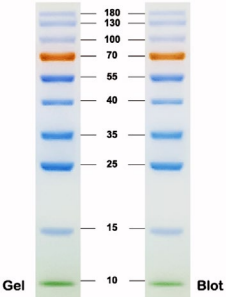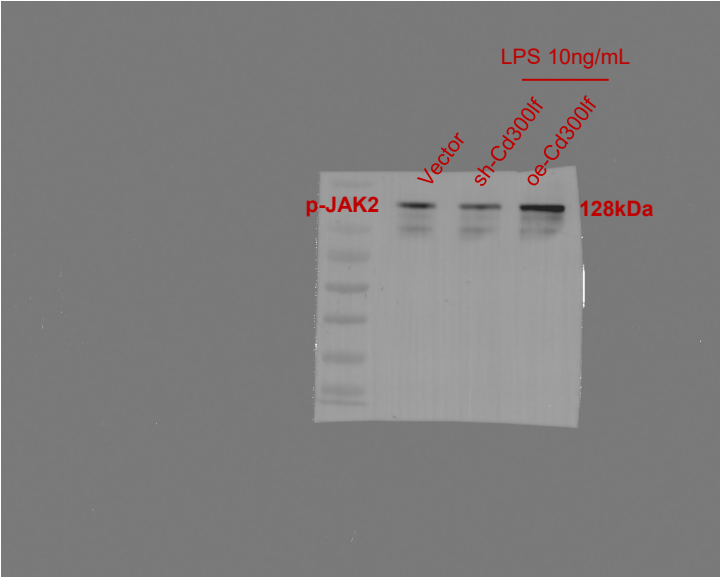

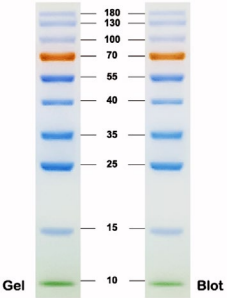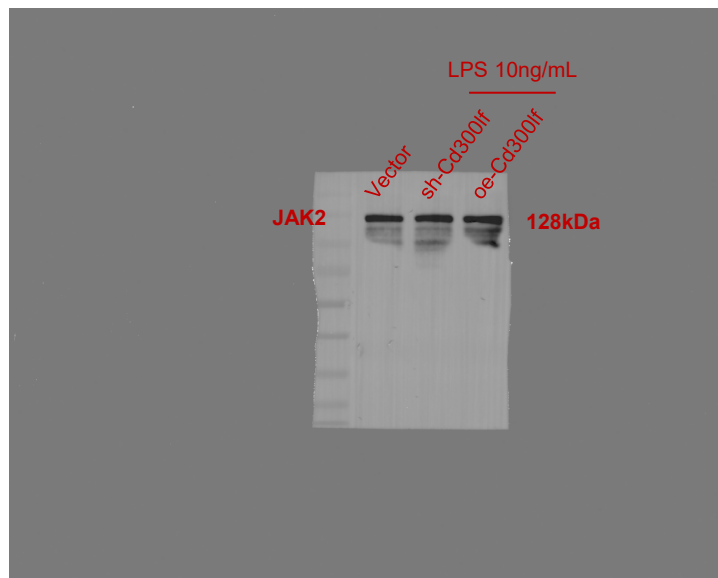

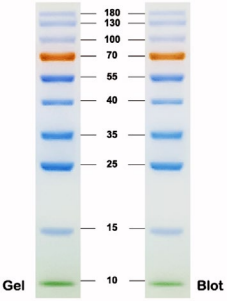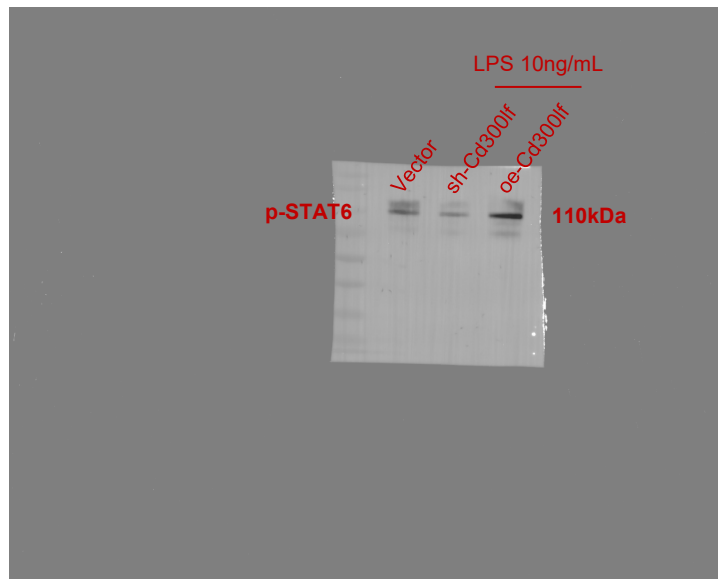

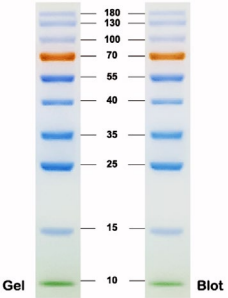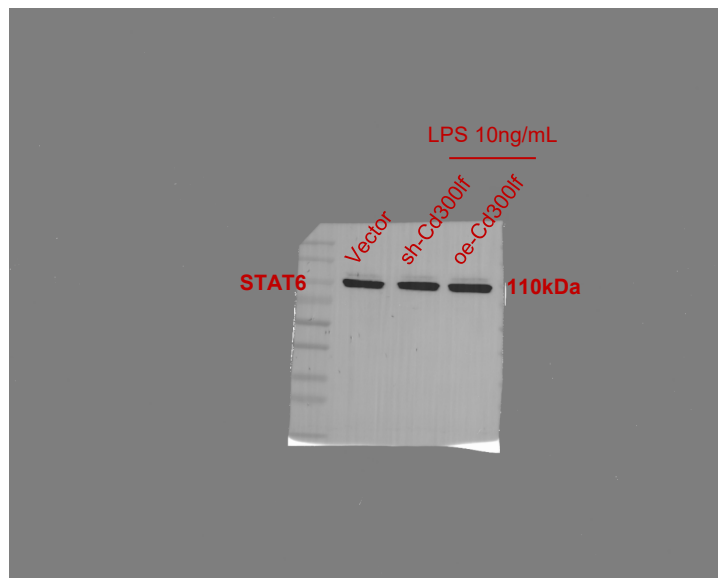

Supplement: Supplementary file 1 — Appendix S1. [file CNS-30-e14824-s001.zip › Supplemental Files.pdf]
